# Hydroxychavicol: A phytochemical targeting cutaneous fungal infections

**DOI:** 10.1038/srep37867

**Published:** 2016-11-29

**Authors:** Intzar Ali, Naresh Kumar Satti, Prabhu Dutt, Rajendra Prasad, Inshad Ali Khan

**Affiliations:** 1 Clinical Microbiology Division, Indian Institute of Integrative Medicine, Jammu Tawi–180 001, India; 2 Membrane Biology Laboratory, School of Life Sciences, Jawaharlal Nehru University, New Delhi–110 067, India; 3 Natural Product Chemistry Division, Indian Institute of Integrative Medicine, Jammu Tawi–180 001, India; 4 Laboratory for Infection Biology, Amity Institute of Integrative Sciences and Health, Amity University, Gurgaon–122 413, India

## Abstract

The present study was designed to investigate the potency of hydroxychavicol on selected cutaneous human pathogenic fungi by the use of *in vitro* and *in vivo* assays and mechanistic characterization along with toxicological effects. Hydroxychavicol consistently displayed a fungicidal effect against all fungal species tested. Inoculum concentrations over the range of 10^4^ to 10^7^ CFU/ml did not significantly alter its antifungal potential and time–kill curve results revealed concentration–dependent killing. It also inhibited the growth of biofilm generated by *Trichophyton mentagrophytes* and *Candida parapsilosis* and reduced the preformed biofilms. Hydroxychavicol was highly effective in the treatment, and mycological eradication of an experimentally induced topical infection model of dermatophytosis (tinea corporis) and cutaneous candidiasis in guinea pigs, respectively. The mode of action of hydroxychavicol appears to originate from the disruption of cell membrane integrity. Administration of hydroxychavicol in mice at 500 mg per kg of body weight by orally produced no overt toxicity. The retention capacity of hydroxychavicol *in vitro*, in the presence of keratin has attributed to its *in vivo* effectiveness in the guinea pig model of topical infections. Furthermore, it is suggestive of its potential use as phytochemical for topical use in cutaneous fungal infections.

Superficial cutaneous fungal infections are one of the most common infectious diseases affecting 20–25% of the general human population worldwide^1^. They are caused by yeasts (e.g., *Candida* species and *Malassezia* species), dermatophytes, and non–dermatophyte species of filamentous fungi (dermatomycoses), which are very common dermatologic conditions and are often resistant to current antifungal treatments, leading to recurrence^2^. This constitutes an important global public health problem as yet unresolved. These infections are caused primarily by a group of filamentous keratinophilic fungi known as dermatophytes that use keratin as a source of nutrient during skin, hair, and nail infection in both immunocompetent as well as immunocompromised individuals, and they also cause invasive infections in immunocompromised patients^3^. Although, it does not cause mortality, however, it does cause significant morbidity and poses a major public health problem especially in the tropical and subtropical regions of countries like India, due to the hot and humid climate^4^. Dermatophytes account for the leading (90%) cases of onychomycosis (fungal nail infections) in the United States and Europe^5^ as well as in India^6^. Onychomycosis (also known as tinea unguium) is a chronic and progressively recurrent fungal infection of the nail designated by nail discoloration, thickening, destruction and deformity^7^. It can be caused by dermatophytes, non–dermatophytes, and yeast^5^ and represents up to 50% of all nail problems and 30% of all cases of dermatophytoses^8^, and the species’ prevalence in fungal nail infections varies with geographical location, climate, and migration^8^,^9^, as well as other epidemiological factors (i.e., age, sex). Cutaneous candidiasis is an infection of the skin that is generally caused by *Candida albicans* and that can be either acute or chronic in nature^10^. However, infections due to non–*albicans Candida* species have emerged over the past two decades, and a shift from *C. albicans* to other species such as *C. parapsilosis*, *C. glabrata*, *C. guilliermondii* and *C. tropicalis* has occurred^11^. Recently, it is also reported that *C. parapsilosis* to be an emergent cause of nail infections^11^. *C. parapsilosis* is the most frequently isolated yeast in the subungual space of the skin of hand in healthy subjects and has been associated with around 50% of *Candida* nail infections and mixed yeast–dermatophyte infections and is also regarded as an important emerging nosocomial pathogen^12^.

Topical therapy has been recently proposed to treat recalcitrant dermatophytosis and onychomycosis as well as other nail disturbances without subsequent occurrence of relapses and re–infections^13^. Topical treatment avoids the systemic effects of oral antifungal therapy, and may be preferred by physicians and patients. Topical agents approved for the treatment of mild to moderate fungal nail infection without matrix involvement include formulations based on ciclopirox (a hydroxypiridone) or amorolfine (a morpholine) or terbinafine (an allylamine)^14^. Ciclopirox, itraconazole, and terbinafine are approved in the United States and around the world for onychomycosis treatment, while amorolfine and fluconazole are approved in Europe^9^. However, a complete cure cannot be expected in each and every case. In contrast, most of the existing topical antifungal has a high affinity to keratin as shown by its *in vitro* experiments, which renders them less active in the horny layer of the epidermis. This property seems to be the major reason why existing drugs are incapable of eradicating the dermatophytes infecting skin tissues^15^.

Therefore, there is still clearly an unmet medical need for new and more effective antifungal chemotherapeutic agents or approaches for treating superficial cutaneous fungal infections. The agents can be applied topically to reduce the limitations associated with the existing drugs. In this regard, natural products, specifically, plant sources have been the single most successful resource for the discovery of new pharmaceuticals. Indeed, various active principles of herbal origin have been shown to exhibit curative effects and are thus marketed as treatments for numerous skin ailments^16^.

*Piper betle* L., (Piperaceae) is a widely distributed plant in the tropical and subtropical regions of the world and the leaves of this plant have been used for a long time in many Asian countries like India and China in the preparation of traditional herbal remedies for a variety of diseases. It has various pharmacological activities such as antimicrobial^17^,^18^, antibacterial^19^, antifungal^20^,^21^,^22^,^23^,^24^,^25^, anthelmintic, antimutagenic, antioxidant, anticarcinogenic, antiinflammatory, gastroprotective, hepatoprotective, immunomodulatory, antiallergic, antipruritic^26^, antiseptic and anticontraceptive^21^ effects. Inspite of its broad clinical applications, few studies have investigated the active components of *P. betle*. Among them, hydroxychavicol is the major phenolic component that belongs to relatively new class (allylbenzene) of natural compounds which has remarkable therapeutic potential. In our earlier report, we explored the *in vitro* antifungal activity of hydroxychavicol^27^. The present study is aimed at in depth characterization of hydroxychavicol for its inhibitory potential against dermatophytes alongwith its mechanism of action.

## Results

### *In vitro* study

#### Antifungal susceptibility results

##### MIC and MFC

It was consistently observed that all tested fungal species (One reference strain and nine clinical isolates of each five species) were susceptible to hydroxychavicol and exhibited MIC ranged from 8 to 32 μg/ml (geometric mean [GM], 19.2) for *Epidermophyton floccosum*, 16 to 32 μg/ml (GM, 27.2) for *Microsporum gypseum*, 8 to 32 μg/ml (GM, 25.6) for *Trichophyton mentagrophytes*, 16 to 64 μg/ml (GM, 30.4) for *Trichophyton rubrum*, and 32 to 64 μg/ml (GM, 44.8) *Candida parapsilosis*, respectively (Table 1). The MFC was found to be either the same or only 2–fold greater than the MIC, which indicates that hydroxychavicol has fungicidal potential against all tested species. Among all the fungal species tested, *E. floccosum* was found to be the most susceptible species to hydroxychavicol, their GM MIC and MFC was 19.2 and 26.4 μg/ml respectively followed by *T. mentagrophytes* (GM MIC and MFC was 25.6 and 27.2 μg/ml, respectively). Moreover, *C. parapsilosis* was the least active to hydroxychavicol, their GM MIC and MFC was 44.8 and 51.2 μg/ml, respectively. Terbinafine served as the standard drug control and it was observed that all tested clinical isolates were susceptible (data not shown). DMSO (1%, vehicle control) had no inhibitory effect on the growth of the tested fungal species when compared with the untreated growth control. There were no differences in susceptibility patterns of clinical isolates obtained from different geographical location of India. Reproducible MIC and MFC results were obtained for hydroxychavicol when using one reference strain of each species in a series of susceptibility assays conducted over the entire study period.

##### MIC and MFC in the presence of keratin powder and fetal bovine serum

The activity of some of the drugs reduces in the presence of keratin or serum, probably through drug–protein interactions. So, to predict the antifungal potency of hydroxychavicol in infected skin tissues, we investigated the effect of keratin and serum on the *in vitro* antifungal activity of hydroxychavicol towards cutaneous fungal pathogens was measured by the CLSI reference broth microdilution method. Table 2 and Table 3 show the MIC and MFC of hydroxychavicol for one reference strain of each selected dermatophytes as well as *C. parapsilosis* in RPMI broth medium (CLSI) with and without 5% defatted–keratin powder (Table 2) and 10% inactivated–fetal bovine serum (Table 3), respectively. It was observed that the inhibitory antifungal potential of hydroxychavicol was not affected in the presence of keratin or serum except for *M. gypseum* which showed an MFC 2–fold greater than the MIC. Terbinafine served as the standard drug control in these experiments and the MIC of terbinafine increased by the addition of keratin (8–32 folds) or serum (4–16 folds) against all tested strains, likely due to the high degree of protein binding.

##### Affinity to keratin powder and fetal bovine serum

To clarify the inhibitory antifungal potential of hydroxychavicol in the presence of keratin as well as serum, we examined its affinity to 5% defatted–keratin powder and 10% inactivated–fetal bovine serum in normal sterile saline was measured by the conventional agar well diffusion method as per CLSI guidelines using Mueller–Hinton agar with 2% glucose and methylene blue for *T. mentagrophytes* ATCC 9533 and *C. parapsilosis* ATCC 200954, respectively at a final inoculum concentration of approximately 5 × 10^5^ CFU/ml of each. Hydroxychavicol showed moderate rates of binding to keratin (44.2%) and serum (40.9%) when compared with the unsupplemented control (*P* < 0.05) and probably for this reason its fungicidal effect in the presence of keratin or serum was reduced 2–fold in comparison with its antifungal potential in the absence of its. In these protein binding experiments, terbinafine served as the standard drug control and keratin binding (90–94%) as well as serum binding (88–92%) was observed in both tested strains, likely due to the high degree of protein binding.

##### Inoculum effect

It is well–known that various antifungal agents markedly reduce their *in vitro* activities which are directly proportional to the increasing inoculum size and it is also dependent upon the organism and antifungal agent being tested. Here, in order to investigate the influence of different inoculum sizes on MIC and MFC of hydroxychavicol towards the reference strains of *T. mentagrophytes* ATCC 9533 as well as *C. parapsilosis* ATCC 200954 were studied by the broth microdilution method. Four inoculum sizes were studied: approximately 2.5 × 10^4^ CFU/ml to 2.5 × 10^7^ CFU/ml. Inoculum concentrations over the range of 10^4^ to 10^7^ CFU/ml did not significantly alter the MIC and MFC of hydroxychavicol for both *C. parapsilosis* ATCC 200954 as well as *T. mentagrophytes* ATCC 9533, respectively in RPMI broth (Table 4). At inoculum concentrations of approximately 2.5 × 10^4^ CFU/ml to 2.5 × 10^6^ CFU/ml, the similar MICs were observed for both strain (31.25 μg/ml for *T. mentagrophytes* and 62.5 μg/ml for *C. parapsilosis*, respectively). At an inoculum of approximately 2.5 × 10^7^ CFU/ml, the MIC was 62.5 μg/ml for *T. mentagrophytes* and 125 μg/ml for *C. parapsilosis*, respectively. The MFC was found to be either the same (up to 10^5^ CFU/ml) or only 2–fold greater than the MIC at an inocula concentration of 10^6^ CFU/ml and 10^7^ CFU/ml of each, which indicates that hydroxychavicol has fungicidal potential against both tested strains. Terbinafine served as the standard drug control (amphotericin B for *C. parapsilosis*) and the inhibitory antifungal potential of these drugs was also not affected statistically over the range of 10^4^ to 10^7^ CFU/ml tested inoculum concentrations.

##### Killing curve studies

**Figure 1:**
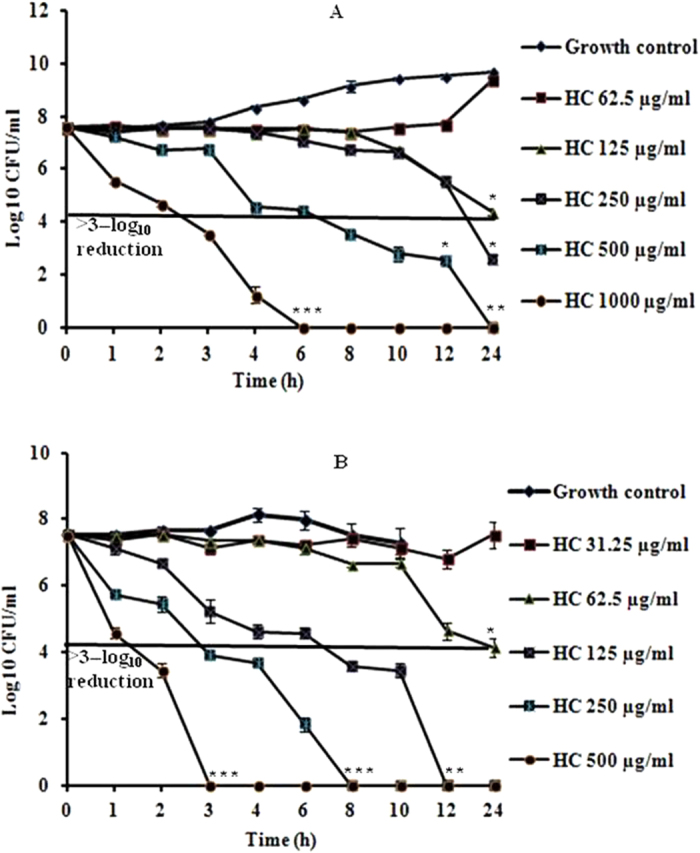
Time–kill curve plots of hydroxychavicol (HC) against *Candida parapsilosis* ATCC 200954 (**A**) and the mixture of germinated as well as ungerminated microconidia of *Trichophyton mentagrophytes* var. *interdigitale* ATCC 9533 (**B**) following exposure of 1 to 16 times the MICs. The cultures were incubated in RPMI 1640 medium buffered to a pH of 7.0 with 0.165 M MOPS buffer (CLSI). The lower limit of accurate and reproducible detectable colony counts was 10 CFU/ml. The solid lines represent a >99.9% growth reduction compared with that of initial inoculums added at the start of the experiment (fungicidal effect). Each time point represents as the mean log_10_ ± standard deviation of two different experiments performed in duplicate with similar results of HC–treated group each time. ^*^P < 0.05; ^**^P < 0.005; ^***^P < 0.0001 (Student’s *t*–test) were considered to be statistically significant when compared with the untreated growth control.

Time–kill curve study is an important parameter to judge the performance of antimicrobial agents. The killing activities of hydroxychavicol for *C. parapsilosis* ATCC 200954 and the mixture of germinated as well as ungerminated microconidia of *T. mentagrophytes* ATCC 9533 at a final cell density of 10^7^ CFU/ml of both species in RPMI broth medium are represented as the mean and standard deviation in Fig. 1(A and B). Hydroxychavicol exhibited fungicidal effect against both strains tested and the reduction in the number of CFU/ml was greater than 3–log_10_ units (>99.9% killing) of the initial inocula added at the start of the experiment. The fungicidal activity was reached at 2 × MIC in 24 h for both strains tested when compared with starting inocula of the untreated growth control (*P* < 0.05). The maximum fungicidal effect with a >5–log_10_ reduction for *C. parapsilosis* was achieved after 24 h as well as 12 h of incubation at 4 × MIC (250 μg/ml) and at 8 × MIC (500 μg/ml) of hydroxychavicol (Fig. 1A). Whereas, at further high concentrations of 8 × MIC (500 μg/ml) and 16 × MIC (1000 μg/ml) the CFU reached below the detection limit of 10 CFU/ml. Interestingly, the fungicidal endpoints below the detection limit of 10 CFU/ml for the mixture of both types of *T. mentagrophytes* microconidia were observed after 12, 8 and 3 h of incubation at 4 × MIC (125 μg/ml), 8 × MIC (250 μg/ml) and 16 × MIC (500 μg/ml) of hydroxychavicol, respectively (Fig. 1B). Regrowth of fungi was also determined in all the treated groups that showed a fungicidal effect below the detection limit of 10 CFU/ml and it was also observed that the killing effect of hydroxychavicol was concentration dependent as well as species dependent. Moreover, measurements of each type of untreated microconidia of *T. mentagrophytes* over time could not be defined accurately because of clumping and the formation of a mycelium, whilst microconidia grown even in the presence of subinhibitory concentration of hydroxychavicol did not undergo clumping and were amenable to plating and counting of colonies. The surviving cells from each experiment recovered after incubation as afore–mentioned and were again tested for *in vitro* susceptibility to hydroxychavicol. These cells exhibited the same MIC (31.25 μg/ml for *T. mentagrophytes*, and 62.5 μg/ml for *C. parapsilosis*) and also exhibited a similar reduction in the number of CFU/ml by hydroxychavicol treated cells during time–kill curve studies. The results of time–kill curve studies with an initial inoculum of 10^7^ CFU/ml, a baseline condition more close to that of the *in vivo* model of experimental topical infections caused by *T. mentagrophytes* ATCC 9533 and *C. parapsilosis* ATCC 200954 in guinea pigs.

##### Biofilm susceptibility results

Hydroxychavicol exhibited an inhibitory (preventative) effect on the biofilm formation and reduction of preformed mature biofilms of *T. mentagrophytes* ATCC 9533 and *C. parapsilosis* ATCC 200954. The results of inhibitory and reduction in the metabolic activities of the biofilms in RPMI broth medium are summarized in Table 5 in the form of mean average optical density and standard deviation along with the percentage of inhibition as treated by hydroxychavicol in comparison with terbinafine as well as amphotericin B, which served as the standard drug control at a concentration of 0.25 × MIC to 8 × MIC of each antifungal agent. The 50% and 80% biofilm inhibitory values of hydroxychavicol was 0.5 × MIC and 1 × MIC, where as the 50% and 80% biofilm reduction values were 1 × MIC and 2 × MIC, respectively. These 50% and 80% inhibitory as well as reduction values of hydroxychavicol were consistently achieved (0.5 × MIC to 2 × MIC) in each time of the experiments. Moreover, reductions of preformed biofilms values were always found to be twofold greater than the concentration required to inhibit biofilm formation at 0.5 × MIC and 1 × MIC of hydroxychavicol. In contrast, its antifungal biofilm potency were observed in stationay phase at the higher tested concentration that were 2 × MIC to 64 × MIC for biofilm inhibition and 4 × MIC to 64 × MIC for biofilm reduction, respectively.

##### *In vivo* efficacy

The therapeutic effect of hydroxychavicol on experimentally induced topical infections caused by *T. mentagrophytes* ATCC 9533 and *C. parapsilosis* ATCC 200954 was examined (Tables 6, 7, 8, [Table t7], [Table t8]). We performed repeated preliminary concentration gradient experiments to define the most influential dose of hydroxychavicol for each strain or to obtain the reproducible results. Eighty times the MIC of hydroxychavicol was selected as the most effective dose for both tested strains in *in–vivo* evaluation study and the results reported here represent the final data obtained in these experiments. The consistently observed MIC of hydroxychavicol against *T. mentagrophytes* ATCC 9533 was 31.25 μg/ml and 62.5 μg/ml for *C. parapsilosis* ATCC 200954 (Tables 3, 4).

##### Therapeutic efficacy of hydroxychavicol against experimental tinea corporis in guinea pigs

An animal passaged *T. mentagrophytes* var. *interdigitale* ATCC 9533 was used for the production of experimental tinea corporis (dermatophytosis) in guinea pigs and the consistently observed CLSI MIC of hydroxychavicol against this strain was 31.25 μg/ml. Table 6 represents fungal burden of the infected skin that was taken two days (48 h) after the last treatment (on day–14 postinfection). *T. mentagrophytes* was recovered from all infected sites of the untreated control as well as vehicle–treated control animals, and yielded the highest average intensity of the skin infection scores (+10). In contrast, when the infected animals were topically treated with 0.25% hydroxychavicol solution (200 μl per loci of infected animal site and two loci per animal) twice–a–day on day–3 (after the 72 h) postinfection and was continued for ten consecutive days, all infected sites of the animals skin were found culture negative.

##### Relapse–preventing effect of hydroxychavicol against experimental tinea corporis in guinea pigs

Table 7 displays the results of the culture study that was carried out nine days after the termination of treatment (on day–21 postinfection). The viable cells of *T. mentagrophytes* ATCC 9533 were recovered from all infected lesions of the untreated control as well as vehicle–treated control animals yielding almost the maximum level of the mean average fungal burden and standard deviation (+9.7 ± 0.48 and +9.8 ± 0.42, respectively), indicating that the infection was continued without spontaneous healing until day–21 postchallenge. On the other hand, the infected animal that was topically treated with 200 μl per loci (16 × MIC) of the 0.25% hydroxychavicol solution twice–a–day for ten consecutive days after three days (72 h) of the postinfection that was showed no occurrence relapse of infection in all the five treated animals.

##### Therapeutic efficacy of hydroxychavicol against experimental cutaneous candidiasis in guinea pigs

A mouse passaged *C. parapsilosis* ATCC 200954 was used for the production of experimental cutaneous candidiasis in guinea pigs and the consistently observed CLSI MIC of hydroxychavicol against this strain was 62.5 μg/ml. The results are presented as the mean and standard deviation of all the infected sites of each group of five animals in Table 8. The viable cells of *C. parapsilosis* were recovered from all infected lesions of the untreated control as well as vehicle–treated control animals yielding high level of the mean average colony counts and standard deviation (log 4.63 ± 0.2 and log 4.98 ± 0.21, respectively) were detected. In contrast, when the infected animals were topically treated with 0.5% hydroxychavicol solution (200 μl per loci of infected animal site and two loci per animal) twice–a–day on day–2 (after the 48 h) postinfection and was continued for five consecutive days, all infected sites of the animals were found to be culture negative (i.e. detection limit, <20 CFU per infected site).

#### Mechanism of action

##### Effect of hydroxychavicol on the yeast cell membrane permeability

As many antimicrobial phenolic compounds are known to exert their effects by altering the membrane permeability of microbes, we attempted to characterize the effects of hydroxychavicol on the integrity of *C. parapsilosis* ATCC 200954 membranes, by monitoring the permeability of the membranes to propidium iodide (PI) and 4, 6–diamidino–2–phenylindole (DAPI), a DNA staining fluorescent probes, into the *Candida* cells. Amphotericin B (AmB) served as the standard drug control in this study.

##### Propidium iodide uptake

**Figure 2:**
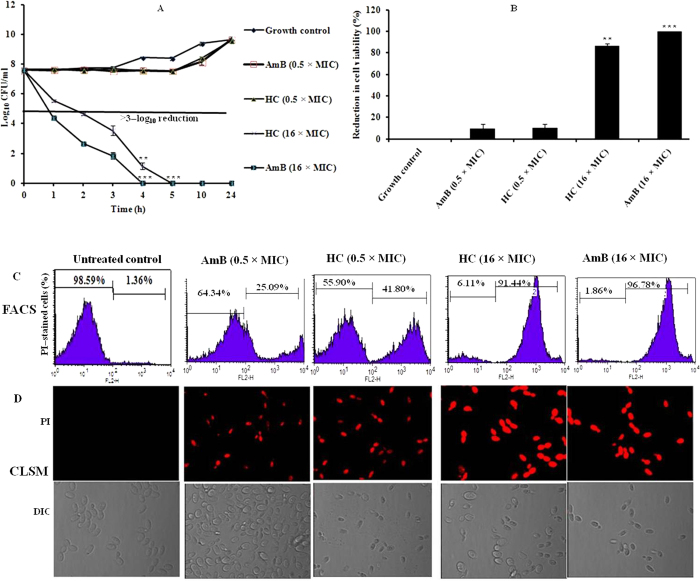
Effect of hydroxychavicol (HC) on the cell membrane permeability of *Candida parapsilosis* ATCC 200954, measured by uptake of propidium iodide (PI). The mid–log growth phase culture at a cell density of approximately 4 × 10^7^ CFU/ml in antibiotic medium 3 buffered to pH 7.0 was exposed to two different concentrations (0.5 and 16 times the MIC) of HC for 4 h at 35 °C and ten minutes prior to the completion of incubation, PI was added at final a concentration of 25 μg/ml. Amphotericin B (AmB) was also tested at a concentration of 0.5 × MIC (sub–inhibitory) and 16 × MIC (fungicidal) as the positive drug control, whilst cells without HC and AmB served as a negative control. The MIC of AmB and HC was 0.25 μg/ml and 62.5 μg/ml respectively at inoculums of approximately 2.5 × 10^4^ CFU/ml^55^. (**A**) Time–kill curve plot of HC and AmB against *C. parapsilosis* ATCC 200954 for 24 h at their indicated MICs, and the percentage reduction in cell viability was calculated after 4 h of exposure (**B**). The percentage of inhibition was calculated with the following equation: 100 – (mean log_10_ CFU per ml of treated × 100/mean log_10_ CFU per ml of untreated growth control). The lower limit of accurate and reproducible detectable colony counts was 10 CFU/ml. The results of three separate experiments with two replicates are represented (means log_10_ ± standard deviations) and similar results were observed each time. ^*^P < 0.05, ^**^P < 0.005, ^***^P < 0.0001 (Student’s *t*–test) were considered to be statistically significant when compared with the untreated growth control. (**C**) Flow cytometric analysis of membrane permeabilization assay by PI uptake. Cells were treated with HC and AmB and stained with PI. After the completion of treatment and staining process, the cellular fluorescence was then analyzed via FACScan flow cytometry. (**D**) Confocal laser scanning microscopy analysis of membrane permeabilization assay by PI uptake in treated as well as untreated yeast cells. The results of selected images are chosen as the best representatives of one of three different experiments with two replicates; similar results were observed each time.

Prior to this assay the cell viability of *C. parapsilosis* ATCC 200954 (≈4 × 10^7^ CFU/ml) following treatment with hydroxychavicol was determined in Penassay broth, and also performed the time course associated with this activity. It was observed that the killing effect of hydroxychavicol was concentration dependent as well as time dependent. A cell viability assay (determined by CFU count) revealed the extent to which the treated cells of *C. parapsilosis* ATCC 200954 were able to survive when removed from exposure to hydroxychavicol (Fig. 2A and B). A 4 h exposure of cells to a subinhibitory concentration of hydroxychavicol (31.25 μg/ml) and AmB (0.125 μg/ml) exhibited a negligible loss in cell viability (≈10%) of each, whereas the 1000 μg/ml (16 × MIC) concentration of hydroxychavicol resulted in a >86% decrease in viability with respect to untreated cells (*P* < 0.05). AmB (standard drug control) was found to be most effective at a concentration of 4 μg/ml (16 × MIC) and produced more than 99.9% loss of cell viability below the detection limit of 10 CFU/ml when compared with starting inocula of the untreated growth control (*P* < 0.0001).

Alteration in membrane permeability is associated with a change in the physical state of the membrane or could be because of compromised cell wall integrity. To determine whether hydroxychavicol treatment induces membrane permeabilization in *C. parapsilosis* ATCC 200954, we measured the uptake of PI, which is a small, cationic, membrane impermeable, fluorescent nucleic acid stain that binds to DNA by intercalating between the bases with little or no sequence preference. Exposing the cell suspension of *C. parapsilosis* (≈4 × 10^7^ CFU/ml) to two different concentrations (0.5 and 16 times the MIC) of hydroxychavicol as well as AmB which served as the standard drug control for 4 h increased cell permeability to PI due to the disruption of membrane integrity as measured by flow cytometry (Fig. 2C) and confocal microscopy (Fig. 2D). These resulted in an increased fluorescence in comparison with the untreated growth controls (Fig. 2C and D).

##### DAPI uptake

**Figure 3:**
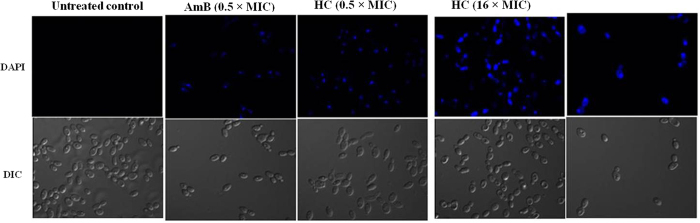
Effect of hydroxychavicol (HC) on the nuclear fragmentation of *Candida parapsilosis* ATCC 200954, measured by DAPI (4, 6–diamidino–2–phenylindole) staining. Cells (≈4 × 10^7^ CFU/ml) of the mid–log growth phase in Penassay broth were incubated for 4 h at 35 °C with an agitation of 200 rpm in a dark incubator chamber in the presence of 0.5 and 16 times the MIC of HC as well as AmB and stained with DAPI at a final concentration of 1 μg/ml as described in the text of mechanism of action. Confocal microscopic images of treated and untreated yeast cells. The captured images are representative of a typical result of one of three independent determinations with two replicates; similar results were observed each time. The MIC of AmB and HC was 0.25 μg/ml and 62.5 μg/ml respectively at inoculums of approximately 2.5 × 10^4^ CFU/ml^55^.

DAPI, a cell–permeable fluorescent dye, is commonly used to detect nuclear morphology changes, including nuclear condensation and fragmentation. It binds strongly and selectively to the minor groove of adenine– and thymine–rich sequence of DNA, thus any increase in fluorescence intensity reflects the extent of DNA damage. DAPI staining of cells treated with hydroxychavicol showed a more split fluorescence intensity within the cells as compared to untreated cells (Fig. 3). The results indicated that hydroxychavicol caused nuclear fragmentation in *Candida* cells.

##### Leakage of 260 nm–absorbing material

**Figure 4:**
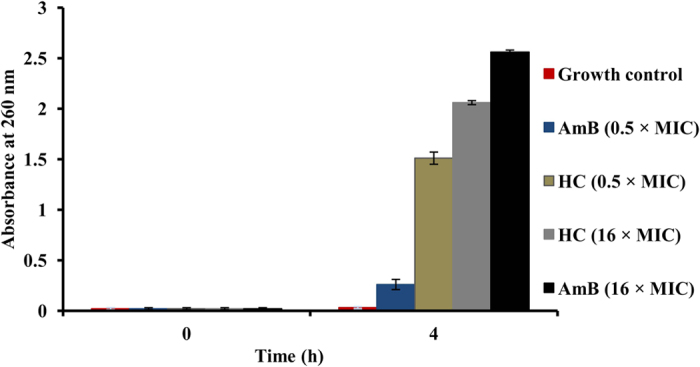
Effect of hydroxychavicol (HC) on *Candida parapsilosis* ATCC 200954, measured by loss of 260 nm absorbing material. The mid–log growth phase culture (≈4 × 10^7^ CFU/ml) in Penassay broth was exposed to HC at a concentration of 0.5 and 16 times the MIC for 4 h at 35 °C with an agitation of 200 rpm in a dark incubator chamber. Treatment with AmB at a concentration of 16 × MIC (4 μg/ml) followed by sonication was used as a positive drug control. The unexposed cell suspension was used as a negative control. The means ± standard deviations of two independent experiments with two replicates are represented and similar results were observed each time. ^*^P < 0.05; ^**^P < 0.001 (Student’s *t*–test) were considered to be statistically significant when compared with the untreated growth control. The MIC of AmB and HC was 0.25 μg/ml and 62.5 μg/ml respectively at inoculums of approximately 2.5 × 10^4^ CFU/ml^55^.

The loss of membrane integrity of the cell suspension of *C. parapsilosis* ATCC 200954 was further confirmed by detection of leakage of 260 nm–absorbing materials. The cell suspension exposed to two different concentrations (0.5 and 16 times the MIC) of hydroxychavicol resulted in increased release of 260 nm–absorbing material, which was comparable to that of AmB at a concentration of 4 μg/ml (16 × MIC) when both were compared with the growth control (*P* < 0.001). AmB at a subinhibitory concentration (0.125 μg/ml) also showed diminished leakage of 260 nm–absorbing materials (Fig. 4) in comparison with growth control (*P* < 0.05).

##### Effect of hydroxychavicol on the yeast cell wall damage

**Figure 5:**
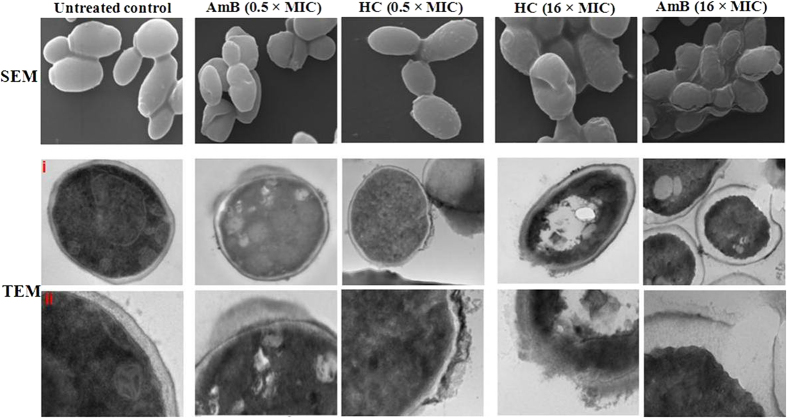
Effect of hydroxychavicol (HC) on the morphology and damage of cell wall of *Candida parapsilosis* ATCC 200954, measured by electron microscopy. Yeast of the mid–log growth phase culture was diluted in Penassay broth with the cell density of approximately 4 × 10^7^ CFU/ml and treated with sub– and supra–MICs of HC as well as AmB (0.5 × MIC and 16 × MIC, respectively) for 4 h at 35 °C with an agitation of 200 rpm in a dark incubator chamber. Scanning electron microscopy (SEM) micrographs and transmission electron microscopy (TEM) micrographs were showing yeast cell wall damage by HC and AmB treated cells at the both concentrations (0.5 × MIC and 16 × MIC). AmB served as the standard drug control, and the unexposed cell suspension was used as a negative control. All images shown were taken at magnifications of 20,000 × for SEM (2 μm) and 10,000 × (i, 100 nm) as well as 30,000 × (ii, 20 nm) for TEM. The selected images are chosen as the best representatives of the experiments. The MIC of AmB and HC was 0.25 μg/ml and 62.5 μg/ml respectively at inoculums of approximately 2.5 × 10^4^ CFU/ml^55^.

To correlate flow cytometry and confocal microscopy data of cell membrane permeability with structural changes of *C. parapsilosis* ATCC 200954 caused by hydroxychavicol and AmB (standard control), scanning electron micrograph data and transmission electron micrograph data were collected for nontreated and treated samples with 0.5 × MIC and 16 × MIC of each in Penassay broth for 4 h at 35 °C as described in the text. Scanning electron microscopy was performed to examine morphological changes of *Candida* cells before and after treatment. The untreated cells exhibited well defined shape with normal smooth surfaces (Fig. 5), and the cell walls of the *C. parapsilosis* that had been incubated with hydroxychavicol as well as AmB caused a series of characteristic alterations, such as surface roughening, disruption, deep wrinkles and deformity along with oozing out of the intracellular content in large quantities. Transmission electron microscopy was employed to study the fungicidal action of treatment. Figure 5 show that untreated cells of *C. parapsilosis* had a uniform cellular architecture with well–defined membranes and no debris in the cell’s surrounding environment. Exposure to hydroxychavicol and AmB resulted in morphological damage, such as loss of the structural integrity of the cell wall, cell membrane, and intracellular matrix. Cell deformation, breakage of cell walls and membranes, condensation of cellular material, and the presence of significant amounts of plasmic material and membrane fragments were observed in the damaged cells of *C. parapsilosis*. We considered this to be a strong indication that the membrane profile had been altered significantly by hydroxychavicol, and points to a critical step of the compound’s candidacidal effects.

##### *In vivo* acute toxicity result of hydroxychavicol in mice

Toxicity studies were performed to determine the maximum tolerable dose of hydroxychavicol after administration of a single–bolus dose ranging from 100 to 1000 mg per kg of body weight by the oral route to overnight fasting Swiss albino mice (n = 10). No adverse pathological symptoms or mortality were observed in the treated animals, and there was no change in general behaviour, when compared with the vehicle–treated group. No death was recorded in the 14 days of the observation period in male or female animals given hydroxychavicol at 500 mg/kg body weight orally. The animals did not show any changes in the general behavior or other physiological activities during the observation period within this concentration tested. In contrast, after given a single–bolus dose of next higher concentration of hydroxychavicol that was 750 mg/kg of body weight orally was found to be toxic (LD_100_). Additionally, the safety of this molecule was also evaluated by using an acute dermal toxicity model in guinea pigs, ranging from 0.1 to 2%. Interestingly, there was no sign of any acute dermal toxicity (primary dermal irritation) in guinea pigs model of the tested concentration.

## Discussion

Despite aggressive therapy employed for the treatment and prophylaxis of superficial cutaneous fungal infections. These are very common dermatologic conditions and are often resistant to current treatments, leading to recurrence^2^. This constitutes an important global public health problem as yet unresolved. Therefore, the identification and development of novel antifungal agents is an important goal of current anti–infective research. In this regard, natural products are attractive prototypes for the search of new entities of plant origin^28^, which provide an unparalleled source of chemical scaffolds with diverse biological activities and have profoundly impacted antimicrobial drug discovery^29^. The potential antifungal effects of certain bioactive compounds from plants have attracted serious attention within the scientific community, largely because of the growing problem of multidrug resistance among pathogenic fungi^30^. Our findings suggest use of plant active principle as potential antifungal agent for topical use in cutaneous mycoses. In traditional Indian medicine, the leaves of *P. betle* have been extensively used as herbal remedies since ancient time. Savaspun *et al.*^31^ first reported the antidermatophyte activity of hydroxychavicol against *T. mentagrophytes*. The study appeared to be preliminary in nature, and described the qualitative activity of hydroxychavicol by using a zone diffusion assay. This prompted us to investigate the fungicidal effect of hydroxychavicol on selected clinically significant cutaneous human fungal pathogens. Prior to our previous study^27^, no detailed antifungal studies had been reported concerning hydroxychavicol, its active compound. Hydroxychavicol has been studied extensively for its various pharmacological activities and selective antifungal efficacy of this phytochemical upthrusts the study furthermore. In this paper, for the first time, we have documented the therapeutic efficacy of hydroxychavicol against experimentally induced topical infection model of cutaneous candidiasis and dermatophytosis (tinea corporis) in guinea pigs along with its effect to prevent relapse after the termination of the treatment. To the best of our knowledge, this is the first report showing that hydroxychavicol possesses antifungal potency *in vitro* and *in vivo* along with its mechanistic characterization against selected cutaneous human pathogenic fungi.

In this study, we aimed to evaluate the *in vitro* antifungal activity of hydroxychavicol against *E. floccosum*, *M. gypseum*, *T. mentagrophytes*, *T. rubrum*, and *C. parapsilosis*, pathogens most commonly associated with onychomycosis. Hydroxychavicol uniformly demonstrated broad–spectrum fungicidal effect against all tested clinically significant cutaneous human pathogenic fungi and consistently with its fungicidal activity, we observed no evidence of trailing endpoints (partial inhibition of growth over an extended range of antifungal concentrations). Interestingly, the fungicidal effect was also most pronounced in dermatophytes including *T. rubrum* (GM MIC and MFC was 30.4 and 48 μg/ml, respectively) which is the etiological agent of more than 70% of all chronic and recurrent clinical infections produced by dermatophytes^4^ suggesting the possibility of using this molecule to inhibit the most prevalent fungus in dermatophytoses. The antifungal activity of hydroxychavicol is largely unknown prior to our earlier study^27^. Moreover, different group of researchers have reported the antimicrobial activity of *P. betle* extract towards various food borne as well as food spoilage microorganisms by using agar diffusion (well and disk), pour plate and agar dilution methods^17^,^18^ and the disagreement between two studies can be due to different methodologies employed. In other studies, the antifungal activity of *P. betle* extract have been reported against various microorganisms like *Alternaria alternata*^20^, *Cladosporium cucumerinum*^21^, *C. albicans*^22^, *Aspergillus niger*, *Aspergillus oryzae* and *Penicillium* species^23^, *M. canis, M. gypseum* and *T. mentagrophytes*^24^, as well as one reference strain of each of the following species of *C. albicans*, *C. dubliniensis*, *C. glabrata*, *C. krusei*, *C. lusitaniae*, *C. parapsilosis*, and *C. tropicalis*^25^.

Dermatophyte species represented by *T. mentagrophytes* and *T. rubrum* parasitize the keratinized tissues of the horny layer of the epidermis, hair, and nails because they can utilize keratin for their growth. Therefore, when antifungal agents are applied to the skin, therapeutic efficiency would depend not only on their antifungal activities but also on their pharmacokinetic properties within the skin tissue^32^. It has been reported that various antifungal agents are markedly reduced in their *in vitro* activities in the presence of keratin or serum, probably due to drug–protein interactions^32^,^33^, which means free drug levels in the body will be significantly lower because of high level of protein binding. The present study demonstrated that the inhibitory antifungal potential of hydroxychavicol was not affected by keratin or serum because of its lower affinity with both of them, suggesting that hydroxychavicol might exist in the infected skin tissues in a free and active form. These favorable biological, physiochemical and pharmacokinetic properties of hydroxychavicol could explain its excellent therapeutic efficacy in the guinea pig models of dermatophytosis (tinea corporis) and cutaneous candidiasis. Moreover, no changes were observed when without defatted–keratin powder and without inactivated–fetal bovine serum, respectively were used. In contrast, the MIC of terbinafine which served as the standard drug control increased by the addition of keratin or serum, likely due to the high degree of protein binding. However, because of the potency of the drug, that increase in MIC may have not adequate clinical implications therefore requires further study. Moreover, terbinafine also showed a high rate of keratin binding (90–94%) as well as serum binding (88–92%), and this observation regarding the activity of terbinafine against *T. mentagrophytes* was in agreement with the findings of Tatsumi *et al.*^33^. It is generally accepted that only the unbound fraction of drug is pharmacologically active, although several studies have demonstrated that increase in MIC for antifungals do not parallel the percentage of protein binding and greater pharmacodynamic effects can be observed in the presence of serum or keratin^34^.

Inoculum concentrations over the range of 10^4^ to 10^7^ CFU/ml did not significantly alter its antifungal potential for *T. mentagrophytes* and *C. parapsilosis*.

Time–kill curves were determined to assess the correlation between inhibitory and fungicidal activity of hydroxychavicol and it was observed that the killing effect of hydroxychavicol toward the reference strains of *C. parapsilosis* ATCC 200954 and the mixture of germinated as well as ungerminated microconidia of *T. mentagrophytes* ATCC 9533 were concentration dependent as well as species dependent. In contrast, Pannu and co–workers^35^ recently reported the time–kill kinetics of the reference antifungal drugs such as ciclopirox, itraconazole, and terbinafine, which are approved in the United States and globally, for onychomycosis treatment^9^ at a concentration of 16 × MIC of each against microconidia and mycelial form of *T. rubrum* in resting conditions (nongrowth) in water by using 10^6^ CFU/ml. Howeover, they observed that these reference antifungal drugs have not shown any significantly reduced colony counts for either dermatophyte form at any time point; an exception was a 3–log_10_ unit reduction by ciclopirox (16 × MIC) after 8 h against nongrowing mycelia, but not microconidia, from *T. rubrum,* inspite of its potent antifungal activity.

Biofilm formation is a very common mode of growth during infection and survival in the environment, and the mature biofilms are notoriously difficult to eradicate and represent a source of infections that are resistant to antifungal. In this context, it was observed that hydroxychavicol was effective in inhibiting the *T. mentagrophytes* ATCC 9533 and *C. parapsilosis* ATCC 200954 generated biofilm with 80% inhibition of biofilm was observed at the MIC concentration and the reduction of preformed mature biofilm was always seen at just only 2–fold greater than the MIC. However, either hydroxychavicol or terbinafine and amphotericin B (AmB), which served as the standard drug control, did not exhibit more than 90% reduction or complete killing (sterility) of the biofilms up to the highest tested concentration of each antifungal agent, which was 4 μg/ml for terbinafine (128 × MIC), 32 μg/ml for AmB (64 × MIC), and 2000 μg/ml for hydroxychavicol in the present study. This observation regarding the activity of AmB against *C. parapsilosis* was in agreement with the findings of Kuhn *et al.*^36^ and Fiori *et al.*^37^. Biofilm–associated microorganisms are refractory to both antimicrobial agents and the host immune responses. It has been reported that the antifungal drug concentrations required to reduce metabolic activity by 50% were 5 to 8 times higher for biofilms than for planktonic cells and 30 to 2000 times higher than the corresponding MICs^38^. Even the newer safe, effective and broader–spectrum antifungal drugs, such as third–generation triazoles and the more expensive echinocandins, as well as liposomal formulations of AmB, have not been able to demonstrate complete eradication of sessile organisms within mature biofilms^36^,^37^.

In the *in vivo* study, we report on the promising therapeutic efficacy of hydroxychavicol against experimentally induced topical infection model of tinea corporis (dermatophytosis) and cutaneous candidiasis in guinea pigs. Hydroxychavicol consistently showed *in vitro* fungicidal effect against *T. mentagrophytes* as well as *C. parapsilosis*, and its correlation with *in vivo* activities, as we observed by the topically treated animals with hydroxychavicol solution. These findings are the first ever report of hydroxychavicol activity in guinea pig models of cutaneous fungal infections. Interestingly, the ointments of the *P. betle* leaves extract have been used for treating and preventing a variety of skin diseases in humans^39^. It has also been reported that 1% topical ointments of the *P. betle* leaves extract successfully controlled tinea infection in humans^40^. There have also been several reports of topical formulations of *P. betle* leaf extract in the form of an ointment, that prevents the growth of *Staphylococcus aureus*, β–hemolytic streptococcus group A and dermatophytes, which are all microorganisms that can cause skin infection^39^,^26^. Pongpech and Prasertsilpe^41^ found that *P. betle* gel inhibited growth of dermatophytes that cause ringworm and growth of *Candida* species more effectively than tolnaftate and with a similar inhibitory effect to that of clotrimazole. However, the effect of *S. aureus* inhibition was less than those of gentamicin and oxytetracyclin/polymyxin B ointment^26^. On the other hand, an herbal mouthwash formulation (HiOra; Himalaya Herbal Healthcare, Bangalore, India) containing *P. betle* leaf extracts was successfully used for control of plaque formation in forty eight human volunteers compared with those of well known clinically approved Essential oil mouthwash (Listerine^®^ mouthwash) and Chlorhexidine mouthwash (Chlohex) formulation in a single blind parallel randomized controlled clinical trial^42^. Moreover, it is often quite difficult to compare our *in vitro* and *in vivo* results with those of previous reports because researchers do not mention chemical composition of *P. betle* as well as because of the lack of standardized methodology of susceptibility testing *in vitro* as per CLSI (Clinical and Laboratory Standards Institute). We hypothesized that the remarkable therapeutic potential effect of *P. betle* leaf extracts in various infections of human beings might be due to the presence of hydrochavicol in the extract.

Furthermore, the relapse–preventing effect of hydroxychavicol against experimentally induced topical infection model of tinea corporis (dermatophytosis) in guinea pigs was also examined nine days after the termination of treatment (on day–21 postinfection) in order to study whether complete mycological eradication was achieved or not. The present study with this tinea corporis model demonstrated that 0.25% hydroxychavicol–treated group rendered all the skin infected sites of guinea pigs culture negative on day–9 posttreatment (on day–21 postinfection) as similar with as on day–2 posttreatment (on day–14 postinfection). However, all the animals skin infected sites of both control groups that is vehicle–treated as well as without vehicle–treated (untreated control), indicated that the infection continued without spontaneous healing until on the day–21 postinfection.

Phenolics are thought to induce alterations in cell membrane permeability^43^,^30^ because they are lipophilic in nature and thus preferentially partition from an aqueous phase into membrane bilayers. In our earlier study, we observed an increased uptake of propidium iodide (PI) by *C. albicans* ATCC 90028 cells when exposed to hydroxychavicol thus indicating that the membrane disruption could be the probable mechanism of action of hydroxychavicol^27^. In order to elucidate the detailed mechanism of action of hydroxychavicol on *C. parapsilosis* ATCC 200954, flow cytometry, confocal microscopy, electron microscopy, and inhibition of ergosterol synthesis studies were performed.

Flow cytometric and confocal microscopy analysis of the hydroxychavicol treated cells revealed enhanced uptake of PI from these cells, indicating that the decrease in viability was accompanied by an increase in cell membrane permeability, which means that dye was very fast penetrated into majority of the yeast cells and the fungicidal influence might have resulted from an extensive lesion within the cell membrane. Therefore, it can be concluded that the observed concentration–dependent fungicidal effect of hydroxychavicol on *C. parapsilosis* is due to direct damage to the cell membrane. Importantly, the effect of hydroxychavicol on the cell membrane was shown to be approximately similar to that resulting from treatment with AmB, which served as the standard drug control, binds to ergosterol, one of the cell membrane sterols, and damages the cell membrane directly, leading to fungicidal activity. We also found that PI uptake did not correlate completely with cell death and this observation regarding the activity staining potential of AmB against *C. parapsilosis* was in agreement with the findings of Ramani and Chaturvedi^44^. In our previous study, it was also observed that uptake of this dye was antifungal concentration dependent as well as yeast species dependent and this was more prominent in the case of AmB^28^.

The marked leakage of cytoplasmic material is thought to be an indication of irreversible membrane damage. Many antimicrobial compounds which act on the bacterial as well as yeast cytoplasmic membrane induces the loss of 260–nm absorbing material, such as thymol, eugenol^45^, phenol derivatives, chlorhexidine^43^,^30^, β–pinene, lemongrass oil, and tea tree oil^46^. During analysis of the absorbance at 260 nm, it was found that *C. parapsilosis* suspensions treated with hydroxychavicol has shown considerable lost of 260 nm–absorbing material, suggesting that nucleic acids were lost through a damaged of plasma membrane. It is also interesting that hydroxychavicol induced cell leakage is approximately similar as compared to that induced by AmB^47^.

Together, our data shows that hydroxychavicol in *C. parapsilosis* causes direct damage to the cell wall and loosening of membrane permeability, which finally leads to cell death by leakage of the cytoplasmic content of the cells. Overall, the increased uptake of PI, leakage of 260 nm–absorbing material, nuclear morphology changes (DAPI staining), and electron microscopy data in the hydroxychavicol treated cells of *C. parapsilosis* in our study, further confirmed the earlier findings that hydroxychavicol alters the cell membrane structure, resulting in the disruption of the permeability barrier of microbial membrane structures^19^. In this study, we found that the cell membrane became permeable to PI, when cells were exposed to hydroxychavicol even at subinhibitory concentrations and AmB served as the standard drug control.

Furthermore, we found extensively altered cell surface morphology and cell wall ultrastructure of *C. parapsilosis* after hydroxychavicol treatment, using scanning electron microscopy and transmission electron microscopy studies and this observation was in agreement with the findings of Nalina and Rahim^19^. The cell membrane disrupting effect of crude extract of *P. betle* leaves containing 39.31% of hydroxychavicol on *Streptococcus mutans* have been demonstrated through the transmission electron microscopy findings by Nalina and Rahim^19^. They found that this *P. betle* leaves extract which causes plasma cell membrane damage and coagulation of the nucleoid could be due to the fatty acids and hydroxy fatty acid ester components present in the extract. The hydrophobic parts of the compound may enable them to partition the lipids of the bacterial cell membrane, thereby disturbing the structures and rendering them more permeable. When the membrane is more permeable, other components present in the extract could make its way into the bacterium and coagulate the nucleoid while maintaining the cell intact^19^. This opinion is strongly supported by our hydroxychavicol data from this study and previous studies demonstrating changes in permeability and increases in membrane fluidity after treatment with geraniol^48^, thymol and eugenol^45^, and tea tree oil^46^.

The antimicrobial trait of hydroxychavicol appears to affect membrane properties and integrity in a manner consistent with other lipophilic, broad–spectrum, membrane–active agents, such as geraniol^48^, thymol, eugenol^45^, phenol derivatives, chlorhexidine^43^,^30^, and tea tree oil^46^.

Hydroxychavicol did not show any *in vivo* acute toxic effect in mice up to a concentration of 500 mg per kg of body weight by the oral route. The present results showed that hydroxychavicol is safe for acute use *in vivo. In vitro* cytotoxic effect of hydroxychavicol was determined towards normal human breast epithelial cell line (fR2) showing IC_50_ values of more than 500 μM. This observation was in agreement with the earlier report of Meenakshi *et al.*^49^ demonstrating it nontoxic to normal human cell lines and mice. Moreover, several groups have reported the *in vitro* safe toxicity profile of hydroxychavicol. In freshly isolated rat hepatocytes Nakagawa *et al.*^50^ demonstrated that hydroxychavicol did not show any cytotoxic effect when tested up to the 500 μM.

In conclusion, on the basis of the results of *in vitro* study of hydroxychavicol as was presented in our previous paper^27^, and *in vitro*, *in vivo,* and toxicity findings reported in this study indicate that hydroxychavicol might be an effective topical antifungal agent for the treatment of dermatophytosis (tinea corporis) and cutaneous candidiasis. Hydroxychavicol is a promising treatment, achieving mycological eradication without relapse and reinfection, and low toxicity. The findings reported in this study strongly suggest the use of hydroxychavicol as a topical antifungal agent for the treatment of cutaneous fungal infections, as an alternative to currently used topical agents for the treatment and prevention of superficial fungal infections. Specific pharmacological approaches will be needed in future clinical trials to improvise its use as a phytotherapeutic agent. However, currently preclinical studies are underway, to determine the antifungal concentrations and therapeutic potential in the topically treated animals by hydroxychavicol solution, and its correlation between the *in vivo* responses to various other models of human pathogenic fungi with the *in vitro* minimum fungicidal concentrations of hydroxychavicol.

## Materials and Methods

### *In vitro* study

#### Antifungal agents

**Figure 6:**
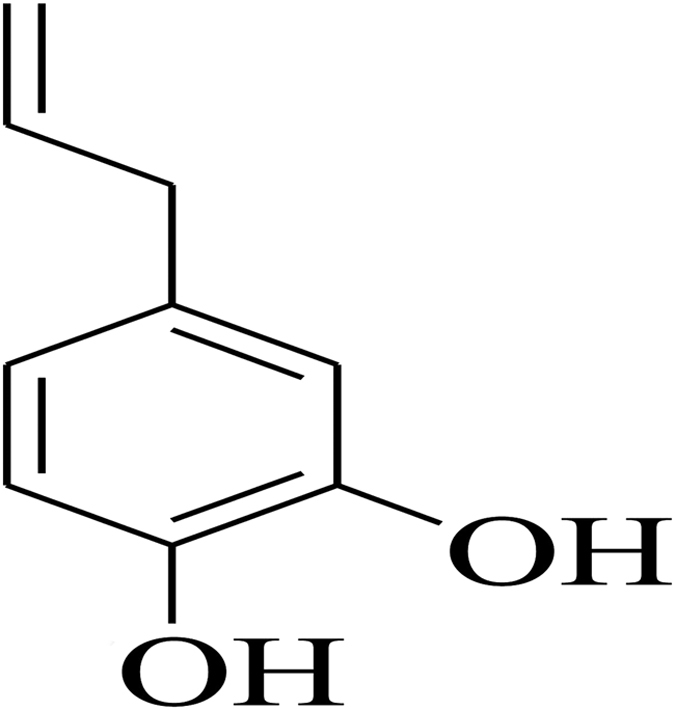
Chemical structure of hydroxychavicol (1–allyl–3, 4–dihydroxybenzene) isolated from *Piper betle* L., (Piperaceae).

Hydroxychavicol (1–allyl–3, 4–dihydroxybenzene) (Fig. 6) was isolated in the pure form from the chloroform extraction of the aqueous leaf extract of *P. betle* L. (Piperaceae) as described in our previous study^51^. Amphotericin B and terbinafine were purchased from Sigma–Aldrich (St. Louis, MO, USA).

#### Organisms

One reference strain and nine clinical isolates of each of the following species were used for *in vitro* susceptibility to hydroxychavicol in this study: *E. floccosum* MTCC 613, *M. gypseum* MTCC 2819, *T. mentagrophytes* ATCC 9533, *T. rubrum* MTCC 296 and *C. parapsilosis* ATCC 200954. Reference strains were procured from the American Type Culture Collection (ATCC, Manassas, VA, USA), and Microbial Type Culture Collection (MTCC, Chandigarh, India). Clinical isolates were randomly selected from our laboratory stock culture collections at the Clinical Microbiology Division of Indian Institute of Integrative Medicine, Jammu Tawi, India, which were obtained from 2007 to 2011 from different hospitals in various parts of India. The collection date for these isolates was not available; moreover the patients and sample details were noted in the records (as allotted registration numbers) of the hospitals. The clinical isolates used in this study were obtained as a gift from Acharya Shri Chander College of Medical Sciences, Sidhra, Jammu, India^27^ and Christian Medical College, Vellore, Tamil Nadu, India. The working strains were maintained by periodic passage on potato dextrose agar (PDA; Difco Laboratories, Detroit, MI, USA) slants at 4 °C and were sub–cultured twice prior to testing to ensure viability and purity.

#### Preparation of inocula

A stock inoculum suspension of each isolate of dermatophytes was prepared from fresh, mature (7– to 10–day–old) cultures grown either on PDA or PDA supplemented with 2% in–house rice flour at 28 °C^52^. The fungal colonies were covered with 3 ml of normal sterile saline containing 0.05% polysorbate–20 (NST; HiMedia Laboratories, Mumbai, India) and the suspensions were made by gently scraping of the surface with a sterile loop generating a mixture of conidia and hyphal elements. These mixtures were filtered with several layers of sterile gauze to remove hyphal elements and agar blocks. The suspension was then transferred to a sterile syringe attached to a sterile filter with a pore diameter of 8 μm (Whatman filter model 40), filtered and collected in a sterile tube. The inoculum size was adjusted to a range of 1 × 10^6^ to 5 × 10^6^ CFU/ml by microscopic enumeration with a cell–counting hematocytometer and then, the optical densities of these suspensions were also measured with a spectrophotometer (Multiskan Spectrum, Thermo Electron, Vantaa, Finland) at a wavelength of 530 nm to a transmittance of 65 to 70% to yield an initial inoculum of 1 × 10^6^ to 5 × 10^6^ CFU/ml^52^. The inoculum suspensions of *C. parapsilosis* was prepared in NST by touching 3 to 5 colonies from 24 h–old cultures grown on PDA plates at 35 °C as per Clinical and Laboratory Standards Institute (CLSI)^53^ guidelines. Inocula were verified for each assay by plating onto PDA plates for colony enumeration.

### Antifungal susceptibility testing assays

#### MIC and MFC determination of hydroxychavicol

The broth microdilution testing was performed in accordance with CLSI documents M38–A2^54^ and M27–A3^53^ by using RPMI 1640 medium with _L_–glutamine, without sodium bicarbonate, buffered to pH 7.0 with 0.165 M 3–(*N*–morpholino) propanesulfonic acid (MOPS) buffer (both from Sigma). Stock solution of hydroxychavicol was prepared in 100% dimethyl sulfoxide (DMSO; Sigma), to a final DMSO concentration of 1% (vol. per vol.) and 2–fold serial dilutions were prepared in RPMI broth medium to yield twice the final concentration required for testing, ranging from 512 to 1 μg/ml. The final inoculum concentration of 0.5 × 10^4^ to 5 × 10^4^ CFU/ml for both dermatophytes^54^ and *C. parapsilosis*^55^. Terbinafine served as the standard drug control. The microtiter plates were incubated at 28 °C for 7 days for dermatophytes^52^, and at 35 °C for 48 h for *C. parapsilosis*^53^. The plates were either read visually with the help of an inverted magnifying reading mirror or with a spectrophotometer. The MIC (minimum inhibitory concentration) was defined as the lowest concentration of the antifungal agent that completely prevented visible growth with respect to the growth control.

The MFC (minimum fungicidal concentration) was determined by seeding the entire volume from each clear MIC well onto PDA plates. To avoid antifungal carryover, the aliquots were allowed to soak into the agar and then were streaked for isolation when dry, thus removing the cells from residual drug source^35^. MFC was defined as the lowest concentration of the antifungal agent that resulted in either no growth or less than 2 colonies (99.9% killing) of the initial inocula added at the start of the experiment, after incubation in similar fashion as afore–mentioned in MIC measurements^55^. All experiments were conducted twice in duplicates on separate occasions with freshly prepared inocula and stock solutions.

#### Affinity of hydroxychavicol to keratin and serum

The affinity experiment of the hydroxychavicol to keratin powder and fetal bovine serum was performed as described by Tatsumi and co–workers^33^ with a slight modification. Briefly, 100 μl sample of the hydroxychavicol solution prepared in 100% DMSO at a concentration of 500 mg/ml was dispensed into 9.9 ml normal sterile saline containing 5% defatted–keratin powder and 10% inactivated–fetal bovine serum in separate Erlenmeyer flask. After agitating at 35 °C for 1 h (100 rpm), the mixture was centrifuged at 3,300 × *g* for 10 min, and five 100 μl portions of the supernatant were taken to determine the rate of binding of hydroxychavicol to keratin and serum, respectively.

The hydroxychavicol concentration in the sample solution was measured by the conventional agar well diffusion method as per CLSI guidelines^56^ by using Mueller–Hinton agar with 2% glucose and methylene blue (HiMedia), medium agar plates (90–mm diameter) containing a final inoculum concentration of approximately 5 × 10^5^ CFU/ml of *C. parapsilosis* ATCC 200954 and *T. mentagrophytes* ATCC 9533 for serum affinity and keratin affinity experiments, respectively. Assay plates were prepared in duplicate. Hydroxychavicol solution at a concentration of 500 mg/ml was serially diluted twofold with 100% DMSO, and finally diluted to 1:100 with normal sterile saline. Wells (6 mm in diameter) were cut in the agar. A 50 μl aliquot of hydroxychavicol diluent was dispensed into the well with the final concentration ranging from 250 to 31.25 μg/ml, and incubated at 35 °C for 24 h for *C. parapsilosis* and at 28 °C for 5 days for *T. mentagrophytes*. In these protein binding experiments, terbinafine served as the standard drug control. Diameters of zones of inhibition (ZOI) were measured with a vernier caliper to the nearest whole millimetre (0.01 mm) at the point where there was a prominent reduction of growth (100%), and the percentage of protein binding was calculated with the following equation: (mean ZOI in the presence of protein – mean ZOI in the absence of protein/mean ZOI in the presence of protein) × 100.

#### Inoculum effect

The MIC and MFC of hydroxychavicol for *T. mentagrophytes* ATCC 9533 and *C. parapsilosis* ATCC 200954 were determined by broth microdilution methods as per the guidelines of CLSI as described above with the final concentration ranged from 1000 to 1.95 μg/ml. Terbinafine served as the standard drug control (amphotericin B for *C. parapsilosis*). Final inoculum concentrations were approximately 2.5 × 10^4^ CFU/ml, 2.5 × 10^5^ CFU/ml, 2.5 × 10^6^ CFU/ml, and 2.5 × 10^7^ CFU/ml.

#### Time–kill curve study

Time versus concentration fungicidal effect of hydroxychavicol on *C. parapsilosis* ATCC 200954 and the mixture of germinated as well as ungerminated microconidia of *T. mentagrophytes* ATCC 9533 in RPMI broth medium (CLSI) was performed as described by Pfaller and colleagues^55^. One milliliter of the adjusted inoculum suspension of *T. mentagrophytes* (≈2 × 10^8^ CFU/ml) was added to 99 ml of medium and incubated for 16 to 18 h in an agitator at 200 rpm at 28 °C for the germination of microconidia^35^. After germination, the hyphae were collected by centrifugation and resuspended in fresh medium, and then this mycelia suspension were mixed with microconidia at a cell density of approximately 2 × 10^7^ CFU/ml. Ten milliliter of these adjusted inoculum suspensions (10^7^ CFU/ml) were dispensed into Erlenmeyer flask and were exposed to with or without hydroxychavicol at a concentration of 1 × MIC to 16 × MIC for the test strain (31.25 to 500 μg/ml for *T. mentagrophytes*, and 62.5 to 1000 μg/ml for *C. parapsilosis*). Culture flasks were incubated in an agitator (200 rpm) at 35 °C for *C. parapsilosis* and at 28 °C for *T. mentagrophytes*. At predetermined time points (0, 1, 2, 3, 4, 6, 8, 10, 12 and 24 h following the addition of hydroxychavicol), a 100 μl aliquot was removed (removed by scraping for *T. mentagrophytes*) from each culture flask and diluted tenfold serially in normal sterile saline containing 0.1% polysorbate–80 (HiMedia) for the inactivation of hydroxychavicol. A 20 μl aliquot was plated onto Sabouraud dextrose agar with soya lecithin and polysorbate–80 (HiMedia), medium agar plate for colony enumeration. When the colony counts were expected to be less than 1,000 CFU/ml, samples of 20 μl or 100 μl were taken directly from the test solution flask and plated or sub–cultured without dilution. Plates were then incubated at 35 °C for 24 to 48 h for *C. parapsilosis* and at 28 °C for 7 days for *T. mentagrophytes.* Importantly in case of *T. mentagrophytes*, the plates were observed daily after 48 h for the presence of growth and the colonies were counted immediately after the observation of visible growth. The lower limit of accurate and reproducible detectable colony counts was 10 CFU/ml. All experiments were conducted twice in duplicate on separate occasions.

### Biofilm susceptibility assays

#### Effect of hydroxychavicol on biofilm formation

The effect of hydroxychavicol on *T. mentagrophytes* ATCC 9533 and *C. parapsilosis* ATCC 200954 biofilm formation was examined by the broth microdilution method similar to MIC assays for planktonic cells^54^,^53^ as discussed earlier. Stock solution of hydroxychavicol was prepared in 100% DMSO with a final concentration of 1% (vol. per vol.) and 2–fold serial dilutions were prepared in RPMI broth medium (CLSI) in amounts of 100 μl per well in 96–well flat–bottom polystyrene microtiter plate (NUNC, Roskilde, Denmark) to yield twice the final concentration required for testing, which ranged from 2000 to 1.95 μg/ml. A 100 μl aliquot from the adjusted inoculum suspension of approximately 2 × 10^6^ CFU/ml in RPMI broth medium was added to each well of the plate, resulting in a final inoculum concentration of approximately 1 × 10^6^ CFU/ml^57^. Terbinafine (4 to 0.0039 μg/ml) and amphotericin B (32 to 0.03 μg/ml) served as the standard drug control in this study, the medium without the agents was used as the non–treated control and the medium alone as the blank control (four wells of each microtiter plate). Following incubation at 35 °C for 48 h (96 h* T. mentagrophytes*), absorbance was recorded at 490 nm to assess the culture growth. The culture supernatants from each well were then decanted and the planktonic cells were removed by washing the wells with sterile phosphate–buffered saline (PBS, pH 7.4; Sigma). The biofilm formation was quantified by XTT reduction assay (see below).

#### Effect of hydroxychavicol on mature biofilm

The effect of hydroxychavicol was also examined on preformed *T. mentagrophytes* ATCC 9533 and *C. parapsilosis* ATCC 200954 biofilm by the method as described previously^57^. Two 100 μl of standardized cell suspensions (≈1 × 10^6^ CFU/ml) in RPMI broth medium was inoculated into selected wells of a 96–well flat–bottom polystyrene microtiter plate. After incubation at 35 °C for 24 h (72 h for *T. mentagrophytes*), the culture supernatant from each well was decanted and the planktonic cells were removed by washing the wells with sterile PBS. Twofold serial dilutions of hydroxychavicol was prepared in RPMI broth medium at a final concentration of 2000 to 1.95 μg/ml required for testing and a 200 μl aliquot of each dilution was added to the selected wells containing the biofilm. The plate was further incubated at 35 °C for 24 h. Amphotericin B and terbinafine served as the standard drug control and was treated in a similar fashion. After incubation, the plates were decanted and washed three times with 200 μl sterile PBS to remove loosely adherent cells. The biofilm reduction was quantified using an XTT reduction assay as described below. Each experiment was performed three times with four replicates on separate occasions with freshly prepared stock solutions and inocula (exponential growth phase culture of *C. parapsilosis* in yeast peptone dextrose broth) in each time and the standardized cell suspension was used immediately.

#### Measurement of biofilm metabolic activity by XTT reduction assay

A semi–quantitative measurement of biofilm was obtained using an XTT (tetrazolium salt; sodium 2, 3–bis–[2–methoxy–4–nitro–5–sulfophenyl]–2H–tetrazolium–5–carboxanilide; Sigma) reduction assay, performed by the method as described previously^57^. Briefly, XTT solution was prepared in PBS (0.5 mg/ml), filter sterilized through a 0.22 μm filter (Millipore, Bangalore, India) and stored at −80 °C until required. Prior to each assay, menadione solution (10 mM prepared in acetone; Sigma) was filtered and mixed with XTT solution (thawed) at a final concentration of 1 μM. XTT/menadione solution (100 μl) was added to each of the pre–washed wells and the plates were incubated in the dark for 2 to 3 h at 35 °C. Following incubation, 75 μl of the solution was transferred to a fresh microtitre plate and the colour change in the solution was measured spectrophometrically at 490 nm using a microtitre plate reader.

### *In vivo* study

#### Animal care and housing

All the animal handling, number of animals as well as experimental protocols employed in the present study were duly approved by the Institutional Animal Ethics Committee (IAEC; study number MLP–1005, December 2008), and the animals were also used as recommended by the “*Guide for the Care and Use of Laboratory Animals*” from the National Research Council of the U.S. National Academies^58^, which fulfils the principles for animal use in India. Male albino guinea pigs weighing 440 to 505 gm used in this study were procured from the institute’s animal house. The experiments were performed with five animals in each group. All animals were individually housed in standard–size polycarbonate cages with controlled conditions of temperature (23 ± 1 °C) and humidity (55 ± 10%) and a 12: 12 h light: dark cycle; they were fed with green vegetables and standard pellet diet (Ashirwad Industries, Chandigarh, India) and filtered water was provided *ad libitum*. Experimental animals underwent an acclimation period for one week prior to use. Animal infection experiments, subsequent handling and housing of the infected animals were carried out in our containment facility.

#### Production of tinea corporis

An animal skin infection model of tinea corporis was induced by a slight modification of the method as described by Ghannoum and colleagues^13^. *T. mentagrophytes* var. *interdigitale* ATCC 9533 passaged twice on guinea pig skin was used in this study. Briefly, the animals were anaesthetized intraperitonealy with an anaesthetic cocktail of 5 mg per kg body weight of xylazine and 40 mg/kg body weight of ketamine (both from Sigma). The anesthesia was also maintained with diethyl ether (Rankem, New Delhi, India). Before inoculation, the hair of both flanks at upper left side and lower right side skin (3– by 3–cm) of each guinea pig were removed by electric hair clipper followed by the application of hair remover cream for complete removal of hair. The skin was slightly abraded with sandpaper making it more susceptible to infection. A 200 μl of inoculum containing approximately 2 × 10^7^ CFU was applied onto each marked area of the skin by using a sterile pipette–tip and rubbed thoroughly with a sterile cotton–tipped swab (day–0 postinfection). The experimental animals were randomly distributed into three groups comprising of five guinea pigs in each group and two flank loci per animal. One group received a vehicle control containing polyethylene glycol 400–ethanol (75:25; vol. per vol.) and a second group received a vehicle control without excipient (untreated control) and third group was treated with 0.25% hydroxychavicol solution, which dissolved in a mixture of polyethylene glycol 400–ethanol (75:25; vol. per vol.). Each guinea pig was topically treated twice–a–day with 400 μl of 0.25% hydroxychavicol solution and 200 μl per loci of infected animal site. Treatment started on day–3 (after the 72 h) postinfection and was continued for ten consecutive days.

#### Therapeutic efficacy

The therapeutic efficacy of hydroxychavicol for tinea corporis was evaluated essentially by the method as reported previously by Tatsumi and co–workers^15^. Culture studies were done to assess the mycological efficacy of the treatment. Two days after the last treatment (on day–14 postinfection), all animals were euthanized under ether anesthesia, and each treated site was wiped thoroughly with a cotton swab containing 70% ethanol. Ten skin blocks were excised from each treated site and each skin block was implanted onto Sabouraud cyclohexamide chloramphenicol agar (HiMedia) plates. Following incubation at 28 °C for 7 days, the skin blocks yielding fungal growth were regarded as culture–positive, and an animal with more than one culture–positive skin block was considered fungus positive. The fungal burden of an infected site was given a score of +0 to +10, according to the corresponding number of culture–positive skin blocks among the 10 skin blocks studied.

To study the relapse preventive effect, a group of guinea pigs were observed for nine days after the last treatment (on day–21 postinfection). The fungal burden was estimates as mentioned above.

#### Production of cutaneous candidiasis

A guinea pig infection model of cutaneous candidiasis was induced by a slight modification of the method as described by Tatsumi and co–workers^32^. Prior to infection, guinea pigs were immunosuppressed by subcutaneous injection of prednisolone (Sigma) at a dose of 30 mg per kg of body weight on the day before and on the day after the inoculation. A hairless area was prepared on the back of animals as aforementioned^13^ and the slightly abrased area of the skin was inoculated with 200 μl of mouse passaged *C. parapsilosis* ATCC 200954 inoculum containing approximately 2 × 10^7^ CFU applied onto each marked area of the skin by using a sterile pipette–tip and rubbed thoroughly with a sterile cotton–tipped swab (day–0 postinfection). The experimental animals were divided into three groups comprising of five guinea pigs in each group and two flank loci per animal as mentioned above and was treated with 0.5% hydroxychavicol solution, which dissolved in a mixture of polyethylene glycol 400–ethanol (75:25; vol. per vol.). Each guinea pig was topically treated twice–a–day with 400 μl of 0.5% hydroxychavicol solution and 200 μl per loci of infected animal site. Treatment started on day–2 (after the 48 h) postinfection and was continued for five consecutive days.

Culture studies were done to assess the mycological efficacy of the treatment. Two days after the last treatment (on day–8 postinfection), all animals were euthanized with the over–dose of anesthetic ether, and each treated site was wiped thoroughly with a 70% ethanol swab. The skin was excised from each treated site, minced with scissors, homogenized in 4 ml of normal sterile saline containing 0.1% polysorbate–80 (NST; HiMedia) with homogenizer (IKA Ultra Turrax T25 Homogenizer) and 2–fold serial dilutions of the homogenate were prepared in NST. A 200 μl aliquot of each dilution was spread onto Sabouraud dextrose agar with soya lecithin and polysorbate–80 (HiMedia), medium agar plate for colony enumeration as well as also plated onto the CHROMagar (Becton–Dickinson, Cockeysville, MD, USA) and *Candida* diagnostic agar^59^ plates to ensure purity of the strain. Following incubation at 35 °C for 48 h, the number of CFU was enumerated, and the logarithm of the number of CFU per infected site was calculated. The lower limit of accurate and reproducible quantitation was 20 CFU per infected site of treated animals. Each *in vivo* model was induced twice on separate occasions with freshly prepared hydroxychavicol solution and inocula (animal passaged) in both times.

### Mechanism of action

#### Cell membrane permeability assays

##### Propidium iodide uptake

Prior to this assay, the cell viability of *C. parapsilosis* ATCC 200954 following treatment with hydroxychavicol was determined by the method as described above in time–kill curve study except that Antibiotic medium 3 (also known as Penassay broth; Difco) buffered to pH 7.0 was used as a test medium.

The disruptive effect of hydroxychavicol on *C. parapsilosis* ATCC 200954 cell membranes was assessed by using hydroxychavicol–mediated propidium iodide (PI, Sigma) uptake, measured by flow cytometry and confocal microscopy. A cell suspension of *C. parapsilosis* in Penassay broth (2 ml, ≈4 × 10^7^ CFU/ml) was exposed to two different concentration (0.5 and 16 times the MIC) of hydroxychavicol for 4 h under agitation (200 rpm) in a dark incubator chamber at 35 °C and ten minutes prior to the completion of incubation, 20 μl of PI dissolved in sterile MilliQ (double–distilled) water was added at final a concentration of 25 μg/ml^60^. Amphotericin B (AmB) was also tested at a concentration of 0.5 × MIC (sub–inhibitory) and 16 × MIC (fungicidal) as the positive drug control, whilst cells without hydroxychavicol and AmB served as a negative control and were treated in a similar fashion to that of PI staining. After incubation with PI, cells were harvested by centrifugation (3,300 × *g* at 4 °C for 10 min), washed thrice and then resuspended in sterile MilliQ water.

For flow cytometric measurements, 50 μl was transferred to a fluorescence–activated cell sorting tube (Becton–Dickinson Biosciences, New Jersey, USA) containing 950 ml sterile MilliQ water. Each tube was analyzed using a FACSCalibur (Becton–Dickinson) with CellQuest Pro software for data acquisition and analysis. For confocal microscopy, a drop of each suspension placed on glass slide covered with glass cover slip was observed with a Olympus FluoView FV1000 (Tokyo, Japan) confocal laser scanning microscope equipped with argon and HeNe lasers for red fluorescence. Each experiment was conducted three times in duplicate on separate occasions with freshly prepared stock solutions.

##### DAPI uptake

The nuclear fragmentation effect of hydroxychavicol on *C. parapsilosis* ATCC 200954 was assessed by 4, 6–diamidino–2–phenylindole (DAPI; Sigma) staining, measured by confocal microscopy. Cell suspensions of *C. parapsilosis* in Penassay broth (2 ml, ≈4 × 10^7^ CFU/ml) were incubated with a two different concentration (0.5 and 16 times the MIC) of hydroxychavicol as well as AmB for 4 h under agitation (200 rpm) in a dark incubator chamber at 35 °C and ten minutes prior to the completion of incubation, 20 μl of DAPI dissolved in sterile MilliQ water was added at final a concentration of 1 μg/ml^61^. AmB served as the standard drug control, whilst cells without hydroxychavicol and AmB served as a negative control and were treated in a similar fashion to that of DAPI staining. After incubation with DAPI, cells were collected by centrifugation (3,300 × *g* at 4 °C for 10 min), washed three times and then resuspended in sterile MilliQ water. A drop of each stained cell suspension put on glass slide covered with glass cover slip was examined with an Olympus FluoView FV1000 confocal microscope equipped with argon and HeNe lasers for blue fluorescence. The experiment was conducted three times, in duplicate on separate occasions with freshly prepared stock solutions.

##### Leakage of 260 nm–absorbing material

A mid–log growth phase culture of *C. parapsilosis* ATCC 200954 in Penassay broth (2 ml, ≈4 × 10^7^ CFU/ml) were exposed to two different concentration (0.5 and 16 times the MIC) of hydroxychavicol for 4 h under agitation (200 rpm) in a dark incubator chamber at 35 °C. After incubation, cells were pelleted by centrifugation (3,300 × *g* at 4 °C for 10 min), washed thrice and then resuspended in sterile MilliQ water. A pretreatment sample was taken, diluted 1 in 10 in MilliQ water, and filtered through a 0.45 μm–pore–size filter (Millipore). The absorbance of filtrates was read using an Analytik Jena Specord UV–250 spectrophotometer (Analytik Jena AG, Jena, Germany) at 260 nm. Cells exposed to AmB at a concentration of 16 × MIC (4 μg/ml) followed by sonication with a probe sonicator (six cycles of 1 min each) served as the positive drug control. The unexposed cell suspension was used as a negative control. The experiment was performed twice in duplicate on separate occasions.

#### Cell wall damage assays

##### Morphological changes

The methodology of electron microscopy used was adapted from that used by Schrand and colleagues^62^ with a little modification. Scanning electron microscopy was performed to examine morphological changes of *C. parapsilosis* ATCC 200954 cells before and after treatment with hydroxychavicol (0.5 and 16 times the MIC) in Penassay broth (10 ml, ≈4 × 10^7^ CFU/ml) for 4 h under agitation (200 rpm) in a dark incubator chamber at 35 °C. AmB served as the standard drug control. Treated and untreated *C. parapsilosis* cells were harvested (3,300 × *g* for 10 min), washed thrice with sterile 0.1 M phosphate buffer (pH 7.2), and cells were then fixed with fresh 2.5% glutaraldehyde for 2 h, post–fixed with 1% osmium tetroxide for 1 h at 4 °C of each fixation, and after each fixation, washed samples were subsequently dehydrated by sequential treatment of 10 min each with 30, 50, 70, 90, 100, and 100% of ethanol. The dehydrated samples were fixed on the aluminum stubs, air–dried in a desiccator after immersion in hexamethyldisilizane, and then coated with a thin layer of gold (Polaron Emitech–SC7640 Sputter Coater, Loughborough, UK). Microscopy was performed with a Carl Zeiss EVO–40 (Jena, Germany) microscope using an accelerating voltage of 20 kV.

##### Fungicidal action

Transmission electron microscopy was employed to study the fungicidal action of hydroxychavicol. *C.* parapsilosis ATCC 200954 cultures, untreated and treated with hydroxychavicol as well as AmB which served as the standard drug control, were pelleted and placed into the primary fixative (2.5% glutaraldehyde for 2 h), thereafter post–fixative (1% osmium tetroxide for 1 h) at 4 °C each time, after each step cells were washed with sterile 0.1 M phosphate buffer (pH 7.2), followed by dehydration with a graded series of ethanol solutions as mentioned above. Next, the samples were rinsed twice with 100% propylene oxide for 10 min of each, followed by infiltration propylene oxide: 1:1 epoxy resin overnight and then 100% epoxy resin twice (overnight each time). Then, the samples were embedded in epoxy resin (Araldite CY212 kit, catalog no. E009; TAAB Laboratories Equipment Ltd.). Ultrathin sections (60–80 nm thickness) were prepared on Leica EM UC6 (Wetzlar, Germany), mounted on carbon coated copper grids and stained with uranyl acetate and lead citrate. Microscopy of the stained samples was performed with a Jeol JEM–2100 F (JEOL, USA) microscope using an accelerating voltage of 120 kV. All reagents used were electron microscopy grade and purchased from well known standard companies.

##### *In vivo* acute toxicity study of hydroxychavicol in mice

The maximum tolerable dose of hydroxychavicol was determined orally. The acute toxicity studies were carried out following the Organization for Economic Co–operation and Development guidelines^63^ after approval from the Institutional Animal Ethics Committee (IAEC; study number MLP–1005, December 2008). Swiss albino mice 6–8 weeks old with a body weight of 20–30 gm in groups of ten mice of both sexes were used obtained from the institute’s animal house and were housed as described above. A stock solution of hydroxychavicol was prepared in 10% ethanol just prior to the experiment and administered to overnight–fasted mice orally with a single–bolus dose in the range of 100 to 1000 mg per kg of body weight. The mice were administered different doses of the test compound and was observed individually after dosing at least once during the first 30 min and periodically for the first 24 h, with special attention given during the first 4 hr and daily thereafter, for a total of 14 days, simultaneously, general behaviour was also observed. The experiment was performed three times, on separate occasions with freshly prepared stock solutions. Additionally, we also tested this molecule for acute dermal toxicity at a concentration of 0.1 to 2% in glycol 400–ethanol (75:25; vol. per vol.) in guinea pigs similar as *in vivo* efficacy experiments.

##### Statistical analysis

All experiments were repeated two to three times with minimum of two replicates for each condition tested and similar results were obtained on all occasions.

##### Tinea corporis

The animals that were culture negative were considered to have less than one skin block showing no growth out of ten blocks per infected site. More than one skin block showing fungus–culture growth out of ten blocks per infected site was considered to be culture positive.

##### Cutaneous candidiasis

The animals that were culture negative were considered to contain below the lower detection assay limit of the number of organisms colonies for numerical and statistical purposes calculated. The frequency of fungus–positive animals and the logarithm of the number of CFU in the infected sites were analyzed. The lower limit of accurate and reproducible quantitation was 20 CFU per infected site of treated animals.

The results are expressed as the mean ± standard deviations (Excel 2003; Microsoft Corp., Redmond, WA, USA), and statistical analysis was carried out using Student’s *t*–test and one–way analysis of variance (ANOVA) for comparison of the multiple means followed by Bonferroni test using GraphPad *Instat2* program (GraphPad Software Inc., La Jolla, CA, USA). *P* values of < 0.05 were considered to be statistically significant.

## Additional Information

**How to cite this article**: Ali, I. *et al.* Hydroxychavicol: A phytochemical targeting cutaneous fungal infections. *Sci. Rep.* **6**, 37867; doi: 10.1038/srep37867 (2016).

**Publisher's note:** Springer Nature remains neutral with regard to jurisdictional claims in published maps and institutional affiliations.

## Figures and Tables

**Table 1:** *In vitro* antifungal activity of hydroxychavicol against 50 isolates of selected cutaneous human pathogenic fungi as determined by broth microdilution method following CLSI guidelines.

Species	Antifungal activity (μg/ml)							
	MIC				MFC			
	Range	GM	MIC_50_	MIC_90_	Range	GM	MFC_50_	MFC_90_
*Epidermophyton floccosum*	8–32	19.2	16	32	16–64	26.4	16	32
*Microsporum gypseum*	16–32	27.2	32	32	32–64	43.2	32	64
*Trichophyton mentagrophytes*	8–32	25.6	32	32	16–32	27.2	32	32
*Trichophyton rubrum*	16–64	30.4	32	32	32–64	48	32	64
*Candida parapsilosis*	32–64	44.8	32	64	32–64	51.2	64	64

CLSI stand for Clinical and Laboratory Standard Institute, MIC stand for minimum inhibitory concentration, MFC stand for minimum fungicidal concentration, and GM stand for geometric mean. One reference strain and nine clinical isolates of each species were tested. The MIC of *C. parapsilosis* was determined by using higher range of inoculum^55^. The 50% and 90% of MICs as well as MFCs, at which 50% and 90% of the isolates tested were inhibited and killed, respectively. The MIC and MFC values of hydroxychavicol against 29 isolates of these species viz. 5 reference strains and 24 clinical isolates were also reported in our previous study^27^. Terbinafine served as the standard drug control and it was observed that all tested clinical isolates were susceptible (data not shown). The results are representative of two separate experiments performed in duplicate and similar results were observed each time.

**Table 2:** Effect of keratin powder (KP) on the *in vitro* antifungal activity of hydroxychavicol against one reference strain of each selected dermatophytes as determined by broth microdilution method following CLSI guidelines.

Species	Antifungal activity (μg/ml)					
	MIC determined in:		Reduced activity (ratio of b/a)	MFC determined in:		Reduced activity (ratio of d/c)
	RPMI^a^ alone	RPMI with 5% KP^b^		RPMI^c^ alone	RPMI with 5% KP^d^	
*E. floccosum* MTCC 613	15.62	15.62	1 fold	15.62	31.25	2 fold
*M. gypseum* MTCC 2819	31.25	31.25	1 fold	62.5	125	2 fold
*T. mentagrophytes* ATCC 9533	31.25	31.25	1 fold	31.25	62.5	2 fold
*T. rubrum* MTCC 296	31.25	31.25	1 fold	31.25	62.5	2 fold

In this experiment, terbinafine served as the standard drug control and the MIC increased by the addition of keratin (8–32 folds) against all tested strains, likely due to the high degree of protein binding (data not shown). The results shown are from one of three separate experiments with two replicates; similar results were observed each time.

**Table 3:** Effect of fetal bovine serum (FBS) on the *in vitro* antifungal activity of hydroxychavicol against one reference strain of each selected cutaneous human fungal pathogens as determined by broth microdilution method following CLSI guidelines.

Species	Antifungal activity (μg/ml)					
	MIC determined in:		Reduced activity (ratio of b/a)	MFC determined in:		Reduced activity (ratio of d/c)
	RPMI^a^ alone	RPMI with 10% FBS^b^		RPMI^c^ alone	RPMI with 10% FBS^d^	
*E. floccosum* MTCC 613	15.62	15.62	1 fold	15.62	31.25	2 fold
*M. gypseum* MTCC 2819	31.25	31.25	1 fold	62.5	125	2 fold
*T. mentagrophytes* ATCC 9533	31.25	31.25	1 fold	31.25	62.5	2 fold
*T. rubrum* MTCC 296	31.25	31.25	1 fold	31.25	62.5	2 fold
*C. parapsilosis* ATCC 200954	62.5	62.5	1 fold	62.5	125	2 fold

The MIC of *C. parapsilosis* was determined by using higher range of inoculum^55^. In this experiment, terbinafine served as the standard drug control and the MIC increased by the addition of serum (4–16 folds) against all tested strains, likely due to the high degree of protein binding (data not shown). The results shown are from one of three separate experiments with two replicates; similar results were observed each time.

**Table 4:** Influence of increasing inoculums size on the *in vitro* antifungal activity of hydroxychavicol against one reference strain of *T. mentagrophytes* var. *interdigitale* ATCC 9533 and *C. parapsilosis* ATCC 200954 as determined by broth microdilution method following CLSI guidelines.

Species	Inoculums size	Antifungal activity (μg/ml)			
		Hydroxychavicol		Drug control	
		MIC	MFC	MIC	MFC
*T. mentagrophytes* ATCC 9533	≈2.5 × 10^4^ CFU/ml^b^	31.25	31.25	0.03	0.06
	≈2.5 × 10^5^ CFU/ml	31.25	31.25	0.03	0.12
	≈2.5 × 10^6^ CFU/ml	31.25	62.5	0.06	0.25
	≈2.5 × 10^7^ CFU/ml	62.5	125	0.12	0.5
*C. parapsilosis* ATCC 200954	≈2.5 × 10^4^ CFU/ml^a^	62.5	62.5	0.5	0.5
	≈2.5 × 10^5^ CFU/ml	62.5	62.5	0.5	1.0
	≈2.5 × 10^6^ CFU/ml	62.5	125	1.0	2.0
	≈2.5 × 10^7^ CFU/ml	125	250	2.0	4.0

CFU/ml stands for colony forming unit per milliliter.

^a^The MIC of *C. parapsilosis* was determined by using higher range of inoculum^55^.

^b^CLSI document M38–A2^54^. Terbinafine served as the standard drug control (amphotericin B for *C. parapsilosis*) and the inhibitory antifungal potential of these drugs was also not affected statistically over the range of 10^4^ to 10^7^ CFU/ml tested inoculum concentrations. The results shown are from one of three different experiments with two replicates; similar results were observed each time.

**Table 5:** Effect of hydroxychavicol on the formation (preventative) and destruction (preformed mature) of *T. mentagrophytes* var. *interdigitale* ATCC 9533 and *C. parapsilosis* ATCC 200954 biofilm as measured by the XTT reduction assay^a^.

Species	Antifungal concentrations	Absorbance: Mean ± SD (% inhibition)			
		Biofilm formation		Mature (preformed) biofilm	
		Hydroxychavicol	Drug control	Hydroxychavicol	Drug control
*T. mentagrophytes*ATCC 9533	0 × MIC	1.48 ± 0.22 (0)	1.48 ± 0.40 (0)	1.59 ± 0.21 (0)	1.59 ± 0.21 (0)
	0.25 × MIC	0.90 ± 0.10* (38.94)	0.75 ± 0.06** (49.08)	1.43 ± 0.12 (9.95)	0.90 ± 0.11* (43.70)
	0.5 × MIC	0.71 ± 0.05** (51.84)	0.59 ± 0.04** (60.28)	1.10 ± 0.09* (30.58)	0.79 ± 0.08** (50.40)
	1 × MIC^b^	0.29 ± 0.03*** (80.32)	0.24 ± 0.02*** (83.48)	0.82 ± 0.04** (48.35)	0.72 ± 0.07** (54.74)
	2 × MIC	0.26 ± 0.01*** (82.04)	0.23 ± 0.03*** (84.47)	0.34 ± 0.01*** (78.72)	0.52 ± 0.09** (66.93)
	4 × MIC	0.22 ± 0.02*** (84.99)	0.20 ± 0.01*** (86.48)	0.27 ± 0.02*** (82.86)	0.43 ± 0.07** (72.52)
	8 × MIC	0.19 ± 0.01*** (86.96)	0.18 ± 0.02*** (87.75)	0.23 ± 0.03*** (84.99)	0.24 ± 0.01*** (84.60)
*C. parapsilosis*ATCC 200954	0 × MIC	0.80 ± 0.10 (0)	0.80 ± 0.10 (0)	0.84 ± 0.11 (0)	0.84 ± 0.11 (0)
	0.25 × MIC	0.53 ± 0.09* (33.43)	0.60 ± 0.11* (24.76)	0.78 ± 0.1 (7.9)	0.80 ± 0.12 (4.75)
	0.5 × MIC	0.41 ± 0.06** (49.36)	0.39 ± 0.08** (51.61)	0.60 ± 0.08* (28.4)	0.58 ± 0.09* (30.89)
	1 × MIC^b^	0.16 ± 0.03*** (79.96)	0.14 ± 0.05*** (82.61)	0.42 ± 0.06** (49.72)	0.45 ± 0.07** (46.61)
	2 × MIC	0.15 ± 0.02*** (80.63)	0.13 ± 0.01*** (83.79)	0.16 ± 0.04*** (80.16)	0.17 ± 0.03*** (79.17)
	4 × MIC	0.14 ± 0.03*** (82.11)	0.13 ± 0.02*** (82.82)	0.16 ± 0.03*** (81.13)	0.15 ± 0.02*** (81.82)
	8 × MIC	0.11 ± 0.02*** (85.82)	0.11 ± 0.01*** (86.05)	0.14 ± 0.04*** (83.30)	0.12 ± 0.01*** (85.05)

^a^Biofilms were grown in 96–well microtiter plates containing RPMI 1640 medium buffered to a pH of 7.0 with 0.165 M 3–(*N*–morpholino) propanesulfonic acid (MOPS) buffer (CLSI) used as assay medium as described in the text of *in vitro* study section. For the prevention assay of biofilm formation, the hydroxychavicol was added at the time of inoculation. For the treatment assay, the hydroxychavicol and fresh medium were added to mature biofilms after 24 h (72 h for *T. mentagrophytes*) of biofilm growth. Inhibition of biofilm formation and destruction of preformed mature biofilm were examined by measuring biofilm metabolic activity (XTT reduction assay) at an absorbance of 490 nm. Terbinafine served as the standard drug control (amphotericin B for *C. parapsilosis*) and was treated in a similar fashion. The values of metabolic activities are expressed as the average optical density (OD) and the percentage of inhibition was calculated with the following equation: 100 − (mean OD_490nm_ of treated well × 100/mean OD_490nm_ of untreated growth control well). The means ± standard deviations (SD) of three separate experiments with four replicates are represented and similar results were observed each time. ^*^P < 0.05; ^**^P < 0.001; ^***^P < 0.0001 (Student’s *t*–test) were considered to be statistically significant when compared with the untreated growth control. ^b^The MIC of terbenafine and hydroxychavicol were 0.03 μg/ml and 31.25 μg/ml, respectively for *T. mentagrophytes*. The MIC of amphotericin B and hydroxychavicol were 0.5 μg/ml and 62.5 μg/ml, respectively at an inoculum of approximately 2.5 × 10^4^ CFU/ml for *C. parapsilosis*^55^.

**Table 6:** Therapeutic efficacy of hydroxychavicol in a guinea pig model of tinea corporis (dermatophytosis)^a^.

Treatment	No. of animals with positive culture/total no. of animals (%)	Average fungal burden of the five infected animals
Untreated control	5/5 (100)	+10.0
Vehicle–treated control	5/5 (100)	+10.0
0.25% hydroxychavicol	0/5 (0)	0

^a^Treatment (200 μl per loci of infected animal site and two loci per animal) was started twice–a–day on day–3 (after the 72 h) postinfection of *T. mentagrophytes* var. *interdigitale* ATCC 9533 and was continued for ten consecutive days. The culture study was carried out two days (48 h) after the last treatment (on day–14 postinfection). The results are representative of two different experiments with similar results each time.

**Table 7 t7:** Relapse of infection in animals receiving treatment with hydroxychavicol in a guinea pig model of tinea corporis (dermatophytosis)^a^.

Treatment	No. of animals with positive culture/total no. of animals (%)	Average fungal burden of the five infected animals
Untreated control	5/5 (100)	+9.7 ± 0.48
Vehicle–treated control	5/5 (100)	+9.8 ± 0.42
0.25% hydroxychavicol	0/5 (0)^**^	0^**^

^a^Treatment (200 μl per loci of infected animal site and two loci per animal) was started twice–a–day on day–3 (after the 72 h) postinfection of *T. mentagrophytes* var. *interdigitale* ATCC 9533 and was continued for ten consecutive days. The culture study was carried out nine days after the last treatment (on day–21 postinfection). The values are expressed as the mean ± standard deviations of two separate experiments and similar results were observed hydroxychavicol–treated group each time. ^**^P < 0.001 (Student’s *t*–test) compared with the control groups.

**Table 8 t8:** Therapeutic efficacy of hydroxychavicol in a guinea pig model of cutaneous candidiasis^a^.

Treatment	No. of animals with positive culture /total no. of animals (%)	Log CFU/infected site (mean ± SD)
Untreated control	5/5 (100)	4.63 ± 0.2
Vehicle–treated control	5/5 (100)	4.98 ± 0.21
0.5% hydroxychavicol	0/5 (0)^*^	ND^*^ (<0.3010)

^a^Treatment (200 μl per loci of infected animal site and two loci per animal) was started twice–a–day on day–2 (after the 48 h) postinfection of *C. parapsilosis* ATCC 200954 and was continued for five consecutive days. The culture study was carried out two days (48 h) after the last treatment (on day–8 postinfection). The lower limit of accurate and reproducible detectable colony counts was 20 CFU per infected site of treated animals. ND stands for not detected up to the lower detection limit. The values are expressed as the mean log_10_ ± standard deviations of two independent experiments and similar results were observed hydroxychavicol–treated group each time. ^*^P < 0.05 (Student’s *t*–test) compared with the control groups.

## References

[b1] Havlickova B., Czaika V. A. & Friedrich M. Epidemiological trends in skin mycoses worldwide. Mycoses 51, 2–15 (2008). 18783559 10.1111/j.1439-0507.2008.01606.x

[b2] Panackal A. A., Halpern E. F. & Watson A. J. Cutaneous fungal infections in the United States: Analysis of the National Ambulatory Medical Care Survey (NAMCS) and National Hospital Ambulatory Medical Care Survey (NHAMCS), 1995–2004. Int. J. Dermatol 48, 704–712 (2009). 19570075 10.1111/j.1365-4632.2009.04025.x

[b3] Peres N. T. A., Maranhão F. C. A., Rossi A. & Martinez–Rossi N. M. Dermatophytes: host–pathogen interaction and fungal resistance. An. Bras. Dermatol. 85, 657–667 (2010). 21152790 10.1590/s0365-05962010000500009

[b4] Venkatesan G., Singh A. J. A. R., Murugesan A. G., Janaki C. & Shankar S. G. *Trichophyton rubrum*–the predominant etiological agent in human dermatophytoses in Chennai, India. *Afr*. J. Microbiol. Res. 5, 009–012 (2007).

[b5] Elewski B. E. Onychomycosis: pathogenesis, diagnosis, and management. Clin. Microbiol. Rev. 11, 415–429 (1998). 9665975 10.1128/cmr.11.3.415 PMC88888

[b6] Kaur R., Kashyap B. & Bhalla P. Onychomycosis – epidemiology, diagnosis and management. Indian J. Med. Microbiol. 26(2), 108–116 (2008). 18445944 10.4103/0255-0857.40522

[b7] Elewski B. E. Onychomycosis. Treatment, quality of life, and economic issues. Am. J. Clin. Dermatol. 1, 19–26 (2000). 11702301 10.2165/00128071-200001010-00002

[b8] Faergemann J. & Baran R. Epidemiology, clinical presentation and diagnosis of onychomycosis. Br. J. Dermatol. 149, 1–4 (2003). 10.1046/j.1365-2133.149.s65.4.x 14510968

[b9] Siu W. J. J. *et al.* Comparison of *in vitro* antifungal activities of efinaconazole and currently available antifungal agents against a variety of pathogenic fungi associated with onychomycosis. Antimicrob. Agents Chemother. 57, 1610–1616 (2013). 23318803 10.1128/AAC.02056-12 PMC3623347

[b10] Watts C. J., Wagner D. K. & Sohnle P. G. Fungal infections, cutaneous. p. 382–388. In: Schaechter M. (ed.), Encyclopedia of Microbiology, 3^rd^ ed., vol 4 © Elsevier, Oxford (2009).

[b11] Bueno J. G. *et al.* *In vitro* activity of fluconazole, itraconazole, voriconazole and terbinafine against fungi causing onychomycosis. Clin. Exp. Dermatol. 35, 658–663 (2009). 19874354 10.1111/j.1365-2230.2009.03698.x

[b12] Ryder N. S., Wagner S. & Letner I. *In vitro* activities of terbinafine against cutaneous isolates of *Candida albicans* and other pathogenic yeasts. Antimicrob. Agents Chemother. 42, 1057–1061 (1998). 9593126 10.1128/aac.42.5.1057 PMC105744

[b13] Ghannoum M. A., Long L. & Pfister W. R. Determination of the efficacy of terbinafine hydrochloride nail solution in the topical treatment of dermatophytosis in a guinea pig model. Mycoses 52, 35–43 (2008). 18498299 10.1111/j.1439-0507.2008.01540.x

[b14] Ghannoum M., Isham N., Henry W., Kroon H. A. & Yurdakul S. Evaluation of the morphological effects of TDT 067 (terbinafine in transfersome) and conventional terbinafine on dermatophyte hyphae *in vitro* and *in vivo*. Antimicrob. Agents Chemother. 56, 2530–2534 (2012). 22354309 10.1128/AAC.05998-11 PMC3346586

[b15] Tatsumi Y., Yokoo M., Arika T. & Yamaguchi H. KP–103, a novel triazole derivative, is effective in preventing relapse and successfully treating experimental interdigital tinea pedis and tinea corporis in guinea pigs. Microbiol. Immunol. 46, 425–432 (2002). 12222928 10.1111/j.1348-0421.2002.tb02716.x

[b16] Corazza M., Borghi A., Lauriola M. M. & Virgili A. Use of topical herbal remedies and cosmetics: a questionnaire–based investigation in dermatology out–patients. J. Euopean Acad. Dermatol. Venereol. 23, 1298–1303 (2009). 10.1111/j.1468-3083.2009.03314.x 19486228

[b17] Jenie B. S. L., Andarwulan N., Puspitasari–Nienaber N. L. & Nuraida L.. Antimicrobial activity of *Piper betle* Linn extract towards food borne pathogens and food spoilage microorganisms. Food microbiology: general, institute of food technologists annual meeting–New Orleans, Louisiana 3(1) (2001).

[b18] Lopez C. M., Nitisinprasert S., Wanchaitanawong P. & Poovarodom N. Antimicrobial activity of medicinal plant extracts against food borne spoilage and pathogenic microorganisms. Kasetsart J. (Nat. Sci.) 37, 460–467 (2003).

[b19] Nalina T. & Rahim Z. H. A. The crude aqueous extract of *Piper betle* L. and its antibacterial effect towards *Streptococcus mutans*. Am. J. Biotechnol. Biochem. 3, 10–15 (2007).

[b20] Misra S. B. & Dixit S. N. Antifungal activity of leaf extracts of some higher plants. *Acta Bot*. Indica 7, 147–150 (1979).

[b21] Evans P. H., Bowers W. S. & Funk E. J. Identification of fungicidal and nematocidal components in the leaves of *Piper betle* (Piperaceae). J. Agric. Food Chem. 32, 1254–1256 (1984).

[b22] Vaijayanthimala J., Anandi C., Udhaya V. & Pugalendi K. V. Anticandal activity of certain south Indian medicinal plants. Phytother. Res. 14, 207–209 (2000). 10815017 10.1002/(sici)1099-1573(200005)14:3<207::aid-ptr564>3.0.co;2-i

[b23] Wanchaitanawong P., Chaungwanit P., Poovarodom N. & Nitisinprasert S. *In vitro* antifungal activity of Thai herb and spice extracts against food spoilage fungi. Kasetsart J. (Nat. Sci.) 39, 400–405 (2005).

[b24] Trakranrungsie N., Chatchawanchonteera A. & Khunkitti W. Ethnoveterinary study for antidermatophytic activity of *Piper betle*, *Alpinia galanga* and *Allium ascalonicum* extracts *in vitro*. Res. Vet. Sci. 84, 80–84 (2008). 17482221 10.1016/j.rvsc.2007.03.006

[b25] Nordin M. A.–F., Harun W. H.–A. W., Razak F. A. & Musa M. Y. Growth inhibitory response and ultrastructural modification of oral–associated candidal reference strains (ATCC) by *Piper betle* L. extract. Int. J. Oral Sci. 1–7 (2014). 24406634 10.1038/ijos.2013.97 PMC3967311

[b26] Wirotesangthong M., Inagaki N., Tanaka H., Thanakijcharoenpath W. & Nagai H. Inhibitory effects of *Piper betle* on production of allergic mediators by bone marrow–derived mast cells and lung epithelial cells. Int. Immunopharmacol. 8, 453–457 (2008). 18279799 10.1016/j.intimp.2007.11.005

[b27] Ali I. *et al.* *In vitro* antifungal activity of hydroxychavicol isolated from Piper betle L. Ann. Clin. Microbiol. Antimicrob. 9, 7 (2010). 20128889 10.1186/1476-0711-9-7 PMC2841090

[b28] Ali I. *et al.* *In vitro* antifungal activities of amphotericin B in combination with acteoside, a phenylethanoid glycoside from *Colebrookea oppositifolia*. J. Med. Microbiol. 60, 1326–1336 (2011). 21474610 10.1099/jmm.0.031906-0

[b29] Jiang B. *et al.* PAP inhibitor with *in vivo* efficacy identified by *Candida albicans* genetic profiling of natural products. Chem. Biol. 15, 363–374 (2008). 18420143 10.1016/j.chembiol.2008.02.016

[b30] Cowan M. M. Plant products as antimicrobial agents. Clin. Microbiol. Rev. 12, 564–582 (1999). 10515903 10.1128/cmr.12.4.564 PMC88925

[b31] Savaspun K. *et al.* Extraction and isolation of antifungal and antibacterial constituents from the Thai betel (*Piper betle* Linn.). Kasetsart University, Bangkok 10900, Thailand (2009).

[b32] Tatsumi Y., Yokoo M., Arika T. & Yamaguchi H. *In vitro* antifungal activity of KP–103, a novel topical derivative, and its therapeutic efficacy against experimental plantar tinea pedis and cutaneous candidiasis in guinea pigs. Antimicrob. Agents Chemother. 45, 1493–1499 (2001). 11302816 10.1128/AAC.45.5.1493-1499.2001 PMC90494

[b33] Tatsumi Y., Yokoo M., Senda H. & Kakehi K. Therapeutic efficacy of topically applied KP–103 against experimental tinea unguium in guinea pigs in comparison with amorolfine and terbinafine. Antimicrob. Agents Chemother. 46, 3797–3801 (2002). 12435679 10.1128/AAC.46.12.3797-3801.2002 PMC132781

[b34] Elefanti A. *et al.* Inhibitory and fungicidal effects of antifungal drugs against *Aspergillus* species in the presence of serum. Antimicrob. Agents Chemother. 57, 1625–1631 (2013). 23318807 10.1128/AAC.01573-12 PMC3623331

[b35] Pannu J. *et al.* NB–002, a novel nanoemulsion with broad antifungal activity against dermatophytes, other filamentous fungi, and *Candida albicans*. Antimicrob. Agents Chemother. 53, 3273–3279 (2009). 19433562 10.1128/AAC.00218-09 PMC2715584

[b36] Kuhn D. M., George T., Chandra J., Mukherjee P. K. & Ghannoum M. A. Antifungal susceptibility of *Candida* biofilms: unique efficacy of amphotericin B lipid formulations and echinocandins. Antimicrob. Agents Chemother. 46, 1773–1780 (2002). 12019089 10.1128/AAC.46.6.1773-1780.2002 PMC127206

[b37] Fiori B. *et al.* *In vitro* activities of anidulafungin and other antifungal agents against biofilms formed by clinical isolates of different *Candida* and *Aspergillus* species. Antimicrob. Agents Chemother. 55, 3031–3035 (2011). 21422210 10.1128/AAC.01569-10 PMC3101406

[b38] Hawser S. P. & Douglas L. J. Resistance of *Candida albicans* biofilms to antifungal agents *in vitro*. Antimicrob. Agents Chemother. 39, 2128–2131 (1995). 8540729 10.1128/aac.39.9.2128 PMC162894

[b39] Boonyaratanakornkit L. *et al.* Activity of betle leaf ointment on skin diseases. Thai J. Pharm. Sci. 15, 277–287 (1990).

[b40] Bhadauria S. & Kumar P. Broad spectrum antidermatophytic drug for the control of tinea infection in human beings. Mycoses 55, 339–343 (2012). 21929713 10.1111/j.1439-0507.2011.02120.x

[b41] Pongpech P. & Prasertsilpe V. The study of antimicrobial activity of *Piper betle* cream and gel against some fungi, yeast and bacteria. J. GPO. 19, 8–22 (1993).

[b42] Singh A., Daing A. & Dixit J. The effect of herbal, essential oil and chlorhexidine mouthrinse on de novo plaque formation. Int. J. Dent. Hygiene. 11, 48–52 (2013). 10.1111/j.1601-5037.2012.00556.x 22583681

[b43] McDonnell G. & Russell A. D. Antiseptics and disinfectants: activity, action and resistance. Clin. Microbiol. Rev. 12, 147–179 (1999). 9880479 10.1128/cmr.12.1.147 PMC88911

[b44] Ramani R. & Chaturvedi V. Flow cytometry antifungal susceptibility testing of pathogenic yeasts other than *Candida albicans* and comparison with the NCCLS broth microdilution test. Antimicrob. Agents Chemother. 44, 2752–2758 (2000). 10991856 10.1128/aac.44.10.2752-2758.2000 PMC90147

[b45] Bennis S., Chami F., Chami N., Bouchikhi T. & Remmal A. Surface alteration of *Saccharomyces cerevisiae* induced by thymol and eugenol. Lett. Appl. Microbiol. 38, 454–458 (2004). 15130138 10.1111/j.1472-765X.2004.01511.x

[b46] Hammer K. A., Carson C. F. & Riley T. V. Antifungal effects of *Melaleuca alternifolia* (tea tree) oil and its components on *Candida albicans*, *Candida glabrata* and *Saccharomyces cerevisiae*. J. Antimicrob. Chemother. 53, 1081–1085 (2004). 15140856 10.1093/jac/dkh243

[b47] Brajtburg J. & Bolard J. Carrier effects on biological activity of amphotericin B. Clin. Microbiol. Rev. 9, 512–531 (1996). 8894350 10.1128/cmr.9.4.512 PMC172908

[b48] Bard M., Albrecht M. R., Gupta N., Guynn C. J. & Stillwell W. Geraniol interferes with membrane functions in strains of *Candida* and *Saccharomyces*. Lipids 23, 534–538 (1988). 3050345 10.1007/BF02535593

[b49] Meenakshi K., Nayak R., Colah R. & Chattopadhyay S. Attenuation of oxidative hemolysis of human red blood cells by the natural phenolic compound, allylpyrocatechol. Free Radical Res. 47(9), 710–717 (2013). 23822150 10.3109/10715762.2013.816847

[b50] Nakagawa Y., Suzuki T., Nakajima K., Ishii H. & Ogata A. Biotransformation and cytotoxic effects of hydroxychavicol, an intermediate of safrole metabolism, in isolated rat hepatocytes. Chemico–Biological Interact. 180, 89–97 (2009). 10.1016/j.cbi.2009.02.003 19428348

[b51] Sharma S. *et al.* Evaluation of the antimicrobial, antioxidant and anti–inflammatory activities of hydroxychavicol for its potential use as an oral care agent. Antimicrob. Agents Chemother. 53, 216–222 (2009). 18573934 10.1128/AAC.00045-08 PMC2612173

[b52] Barros M. E. S., Santos D. A. & Hamdan J. S. *In vitro* methods for antifungal susceptibility testing of *Trichophyton* species. Mycol. Res. 110, 1355–1360 (2006). 17070026 10.1016/j.mycres.2006.08.006

[b53] Clinical and Laboratory Standards Institute. Reference method for broth dilution antifungal susceptibility testing of yeasts, approved standard. *CLSI document M27–A3. Clinical and Laboratory Standards Institute* (Wayne, PA, 2008).

[b54] Clinical and Laboratory Standards Institute. Reference method for broth dilution antifungal susceptibility testing of filamentous fungi, approved standard. *CLSI document M38–A2. Clinical and Laboratory Standards Institute* (Wayne, PA, 2008).

[b55] Pfaller M. A., Sheehan D. J. & Rex J. H.. Determination of fungicidal activities against yeasts and molds: lessons learned from bactericidal testing and the need for standardization. Clin. Microbiol. Rev. 17, 268–280 (2004). 15084501 10.1128/CMR.17.2.268-280.2004 PMC387411

[b56] Clinical and Laboratory Standards Institute/National Committee for Clinical Laboratory Standards. Method for antifungal disk diffusion susceptibility testing of yeasts, approved guideline. *CLSI document M44–A2*. Clinical and Laboratory Standards Institute (Wayne, PA, 2009).

[b57] Pierce C. G. *et al.* A simple and reproducible 96–well plate–based method for the formation of fungal biofilms and its application to antifungal susceptibility testing. Nat. Protocol 3, 1494–1500 (2008). 10.1038/nport.2008.141 PMC2741160 18772877

[b58] National Research Council. Guide for the care and use of laboratory animals, National Academies Press, Washington, DC, USA (1996).

[b59] Cooke V. M. *et al.* New chromogenic agar medium for the identification of *Candida* species. Appl. Environ. Microbiol. 68, 3622–3627 (2002). 12089051 10.1128/AEM.68.7.3622-3627.2002 PMC126815

[b60] Green L., Petersen B., Steimel L., Haeber P. & Current W. Rapid determination of antifungal activity by flow cytometry. J. Clin. Microbiol. 32, 1088–1091 (1994). 8027319 10.1128/jcm.32.4.1088-1091.1994 PMC267192

[b61] Pringle J. R. *et al.* Fluorescence microscopy methods for yeast. Methods Cell Biol. 21, 357–435 (1989). 10.1016/s0091-679x(08)61620-9 2476649

[b62] Schrand A. M., Schlager J. J., Dai L. & Hussain S. M. Preparation of cells for assessing ultrastructural localization of nanoparticles with transmission electron microscopy. Nat. Protocols 4, 744–757 (2010). 10.1038/nprot.2010.2 20360769

[b63] Organization for Economic Co–operation and Development. *OECD guidelines for testing of chemicals, Guideline 425*: Acute Oral Toxicity – Up-and-down procedure (2006).

